# Research on the Application of Nano-Additives in Gel-like Lubricants

**DOI:** 10.3390/gels11070546

**Published:** 2025-07-14

**Authors:** Han Peng, Zihao Meng, Linjian Shangguan, Lei Liu, Can Yang, Lingxi Guo

**Affiliations:** 1 School of Mechanical Engineering, North China University of Water Resources and Electric Power, Zhengzhou 450045, China; mzh000311@163.com (Z.M.); liulei@ncwu.edu.cn (L.L.); yc03030707@163.com (C.Y.); m15515018351@163.com (L.G.); 2 School of Water Conservancy, North China University of Water Resources and Electric Power, Zhengzhou 450045, China

**Keywords:** gel-like lubricant, nano-additives, friction wear, preparation and classification, performance analysis

## Abstract

In the field of mechanical motion, friction loss and material wear are common problems. As one of the essential components for enhancing the lubricating performance of gel-like lubricants, nano-additives leverage their unique physical and chemical properties to form an efficient protective film on friction surfaces. This effectively reduces friction resistance and inhibits wear progression, thereby playing a significant role in promoting energy conservation, emissions reduction, and the implementation of green development principles. This study first introduces the physical and chemical preparation processes of gel-like lubricant nanoadditives. It then classifies them (mainly based on metal bases, metal oxides, nanocarbon materials, and other nanoadditives). Then, the performance of gel-like lubricant nano-additives is evaluated (mainly in terms of anti-wear, friction reduction, oxidation resistance, and load carrying capacity), and the surface analysis technology used is described. Finally, we summarize the application scenarios of gel-like lubricant nano-additives, identify the challenges faced, and discuss future prospects. This study provides new insights and directions for the design and synthesis of novel gel-like lubricants with significant lubricating and anti-wear properties in the future.

## 1. Introduction

With the continuous increase in energy demand, energy scarcity has gradually become a bottleneck restricting development [1]. Energy loss is undoubtedly a significant negative factor affecting the smooth and efficient operation of society in such a large environment. Friction consumption accounts for about one-fifth of the world’s energy consumption. One third of all energy used in transportation is used to overcome friction [2]. Reducing energy loss becomes an urgent issue [3]. In automotive engines, 40% of the total energy is wasted due to mechanical losses, with the piston-cylinder system accounting for 50% of the friction losses and the piston rings accounting for another 70–80% of the friction losses in this system. Inadequate lubrication can trigger component wear, seizing and even catastrophic failure. Much of the energy in mechanical losses is frictional losses, including direct friction between components. Additional wear and tear due to inadequate lubrication, friction of sealing devices, etc. [4]. Friction loss will not only reduce the efficiency of machinery, but also lead to heat and wear of components, shortening the service life of machinery. Thus, in the course of mechanical design and operation, usually take various measures to reduce friction loss [5]. Adding gel-like lubricant to friction points is one of the key ways to reduce friction [6]. In the process of mechanical equipment operation, the lubrication system plays a very important role. Once lubrication is inadequate, it can lead to a number of serious problems [7]. The primary function of lubrication is to form a film of oil on the surface of parts, reducing direct contact between parts and thereby lowering their coefficient of friction [8]. When the lubrication is not sufficient, this oil film can not completely and effectively cover the surface of the parts, and dry friction or boundary friction will occur between the parts [9]. This high-intensity friction will rapidly accelerate part surface wear [10]. With the increasing wear and tear, the microstructure of the part surface is gradually destroyed, and the originally smooth surface becomes rough and uneven [11]. Therefore, it can be said that good lubrication to enhance the energy utilization efficiency of machinery, to alleviate the problem of energy loss in the context of energy scarcity, has an irreplaceable role, 34.4% of machine failures originated from insufficient lubrication, 19.6% from improper lubrication. In other words, About 54% of machine failures are due to lubrication problems.

Grease is a complex mixture composed of base oil, thickener, and additives [12], with varying proportions of each component: Base oil accounts for 80–95% and is the primary component of grease. It is made from petroleum, synthetic hydrocarbons, or vegetable oils, and its properties directly influence the grease’s service life and lubricating performance. Greases can be classified into mineral oil-based, synthetic oil-based, and vegetable oil-based greases. Among these, mineral oil-based greases are the most commonly used due to their high availability, low cost, and good compatibility with additives, while synthetic oil-based greases offer superior performance in terms of oxidation stability and other properties [13]; Thickeners account for 2–15% and serve to increase viscosity, form a three-dimensional framework to adsorb and fix the base oil, and maintain grease stability. They are divided into metal soap-based (including single soap-based and composite soap-based, such as composite lithium-based) and non-metal soap-based (inorganic and organic categories) [14]; Additives account for 0–10% of the formulation, though their small proportion is critical for tribological performance. These include rust inhibitors, antioxidants, etc., which improve performance and extend service life. Among them, anti-wear additives reduce surface compression and lower friction wear.

Traditional grease generally refers to grease prepared using classic formulas and processes, with metal soap thickeners and mineral base oils as the main components. It has a long history of application and mature technology, and is the most common type of grease in the industrial field.

Gel-like grease are another important category of lubricants, characterized by the use of non-soap-based thickening agent systems. These thickening agents primarily include gels and polymers, such as organically modified clays, polyureas, polytetrafluoroethylene (PTFE), silica, and specific high-molecular-weight polymers. These thickeners bind the base oil by forming three-dimensional network structures (such as clay platelet stacking, polymer chain entanglement, or physical/chemical cross-linking), imparting unique gel-like rheological properties to the grease. Unlike soap-based greases, gel-based greases typically exhibit higher thermal stability, better water resistance, or compatibility with specific base oils/additives, making them suitable for high-temperature, vacuum, or specific chemical environments.

Although traditional additives are widely used in the lubrication field, there is still a big difference between their performance and that of nano grease additives. As shown in Table 1. The addition of nano grease additives provides new ideas for the development of traditional additives.

Gel-like lubricants have found widespread application in the field of mechanical lubrication due to their unique properties [16]. From rolling bearings to sliding bearings, from gears to pivots, from couplings to rails, and from pin bushings to sliding contacts, gel-like lubricants are the lubricant of choice for many mechanical equipment components. Advantages include good sealing performance to prevent leakage, as well as corrosion resistance and virtually no maintenance [17]. In the global economy, lubricating grease plays a pivotal role. It is indispensable in ensuring the normal operation of various mechanical equipment, lowering friction and wear during operation, and extending the service life of mechanical equipment [18]. Gel-like lubricants offer better sealing and leak-proof properties than lubricating oils. With the continuous development and progress of modern industry, the demand for lubricants will continue to grow [19]. Since nanomaterials typically exhibit excellent tribological properties, their use in gel-like lubricants to enhance anti-wear and friction-reducing performance has become a research hotspot in the field of lubrication [20].

The remainder of this paper is organized as follows. Section 2 focuses on the preparation process of nano-additives in gel-like lubricants, providing a comprehensive analysis of the two core technologies: physical preparation methods and chemical synthesis methods; Section 3 systematically classifies nano-additives in gel-like lubricants based on their functional characteristics and mechanisms of action; Section 4 delves into the lubrication and anti-wear performance of nano-additives in gel-like lubricants, while summarizing and categorizing the surface analysis techniques employed in performance studies; Section 5 examines the application scenarios of nano-additives in gel-like lubricants from industrial and mechanical perspectives, highlighting their technological innovation advantages through comparisons with traditional lubricants; Section 6 summarizes the research findings, draws conclusions, and, based on the current state of research, outlines future research trends in areas such as performance optimization and application expansion, providing direction for the future development of nano-additives in gel-like lubricants.

## 2. Preparation of Nano-Additives in Gel-like Lubricants

The preparation methods for nano-additives in gel-like lubricants primarily include physical methods (such as ball milling, ultrasonic processing, etc.) and chemical synthesis methods (such as sol-gel, hydrothermal synthesis, chemical reduction, etc.). Different preparation methods have their own advantages and disadvantages in terms of cost, process complexity, yield, and material performance, and should be selected based on actual application requirements.

### 2.1. Physical Preparation

Kamel et al. [21] prepared graphene nanosheets (GNS) by modified Hummer method and investigated the effect of different concentrations (0.5–4 wt%) of GNS on the tribological properties of calcium-based gel-like lubricant. The results showed that the addition of GNS significantly enhanced the wear-reducing and anti-wear properties of the grease, with a 61% reduction in the coefficient of friction and a 45% reduction in the wear scar diameter (WSD). This suggests that GNS, as a grease additive, can significantly improve the tribological properties and has an important potential for application in the field of efficient lubricating materials. Zhou et al. [22] used orthogonal experimental method to prepare calcium fluoride from phosphogypsum and successfully prepared high quality calcium fluoride nanoparticles from calcined phosphogypsum in aqueous solution by direct precipitation method, and it was found that the average particle size of calcium fluoride was around 70 nm. Liu et al. [23] prepared stable graphene water dispersions (polyethylenimine-reduced graphene oxide nanosheets) by improving the Hummer method and water bath method. In steel ball plate friction tests, the water-based lubricant containing 0.05 wt% PEI-RGO demonstrated excellent performance: compared to pure water, the friction coefficient was reduced by 54.6%, and the wear rate was reduced by 45.0%. This superior performance is attributed to the unique protective film formation and repair mechanism of PEI-RGO nanosheets. During friction, PEI-RGO nanosheets fill micro-defects on the worn surface through physical adsorption (the amino groups of PEI can form hydrogen bonds or electrostatic interactions with the metal surface) or chemical bonding, forming a continuous protective film that directly reduces the metal contact area and effectively lowers the friction coefficient. Additionally, the amount of iron oxide on the wear surface was significantly reduced, as the PEI-RGO in the protective film blocks water and oxygen from contacting the metal, inhibiting the formation of iron oxide. The presence of PEI-RGO on the worn surface was detected in experiments, directly proving that the nanomaterials deposit during friction and participate in membrane formation. This indicates that PEI-RGO nanosheets, as water-based lubricant additives, not only significantly reduce friction and wear but also possess excellent surface protection functions. Their unique protective membrane mechanism makes them of significant practical value in environmentally friendly, high-efficiency lubrication systems.

**Figure 1:**
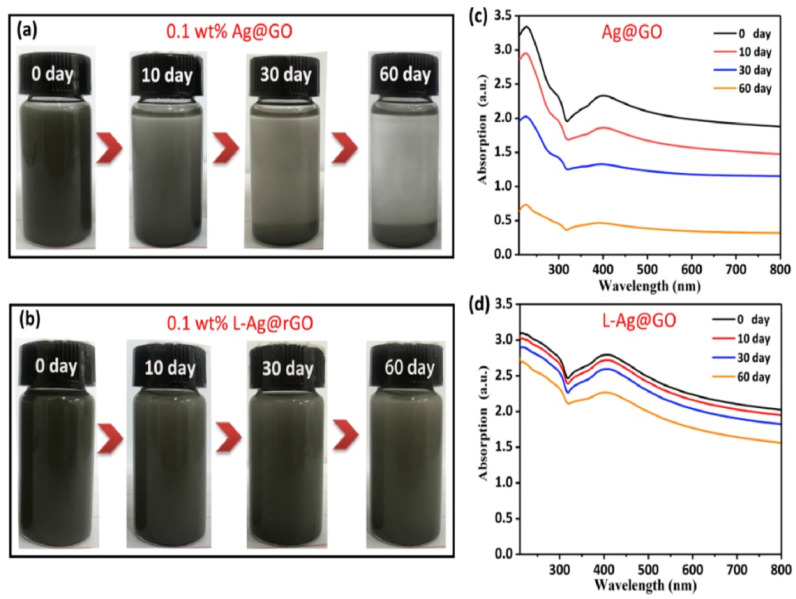
(**a**,**b**) Optical microscope images of pure PL after adding 0.1 wt% Ag@GO and 0.1 wt% L-Ag@rGO at different times. (**c**,**d**) UV-visible absorption spectra of additives in base oil after different settling times. (Reprinted with permission from Reference [25]. Copyright 2020 Elsevier).

Wang et al. [24] used ultrasonic dispersion technology to prepare a lubricant containing gel-like boron nitride (BN) nanoparticles. The study found that BN nanoparticles can form a lubricating film on the friction surface, providing protective effects; simultaneously, their layered structure facilitates sliding at the friction interface. The synergistic effect of the lubricating film and layered structure significantly reduces the friction coefficient, enhancing the anti-friction performance of the grease. This indicates that BN nanoparticles, as grease additives, effectively reduce friction through the synergistic mechanism of lubricating film protection and layered structure sliding, providing a new direction for the development of high-performance lubricating materials. Wang et al. [25] prepared silver/graphene nanocomposites by one-step laser irradiation, which effectively avoided material aggregation and had good dispersion stability. The results showed that the friction coefficient of the composites with the addition of 0.1 wt% was reduced by 40%, and the wear point diameter was reduced by 36% (Figure 1 below).

Jin [26] used co-precipitation, nucleation/crystallization isolation, urea hydrothermal method and regulated the crystallization time to prepare different Layered Double Hydroxides (LDHs). The results showed that the antioxidant capacity of LDHs prepared by urea hydrothermal method was poor. The antioxidant properties of LDHs prepared by the co-precipitation method were gradually weakened with the extension of the crystallization time. The antioxidant properties of LDHs prepared by nucleation/crystallization isolation method showed an increasing trend after crystallization. Lei [27] prepared zinc oxide (ZnO) particles with different morphologies by hydrothermal synthesis and added them to lithium-based, lithium complex and polyurea greases. The experiments showed that ZnO particles significantly enhanced the load-bearing capacity of lithium grease and reduced the coefficient of friction by 32.7% compared with the base grease. ZnO particles as grease additives can effectively enhance the load-bearing performance of the grease and significantly reduce the friction, and they have a great potential to optimize the comprehensive performance of the grease.

Physical preparation methods demonstrate significant process advantages when applied to the production of gel-like lubricant nano-additives. Their operational procedures are straightforward and easy to understand, with low requirements for production equipment, and the process is intuitive and controllable, facilitating large-scale mass production. Certain processes, such as ball milling, are particularly suitable for industrialization due to their low equipment investment and operating costs. However, this method still faces technical challenges in practical applications. Due to the lack of precise control mechanisms, it is difficult to finely regulate the size and morphology of nanomaterials, resulting in a wide particle size distribution range and affecting the uniformity of material properties. Additionally, the preparation process may introduce impurities or structural defects, reducing material purity and performance; nanoparticle agglomeration is highly likely during dispersion, resulting in insufficient dispersion and stability in lubricant systems. To address these issues, the industry often combines nano-additives with gel carriers to improve dispersion performance, enhance stability, and optimize their effectiveness in lubricants.

### 2.2. Chemical Synthesis

**Figure 2:**
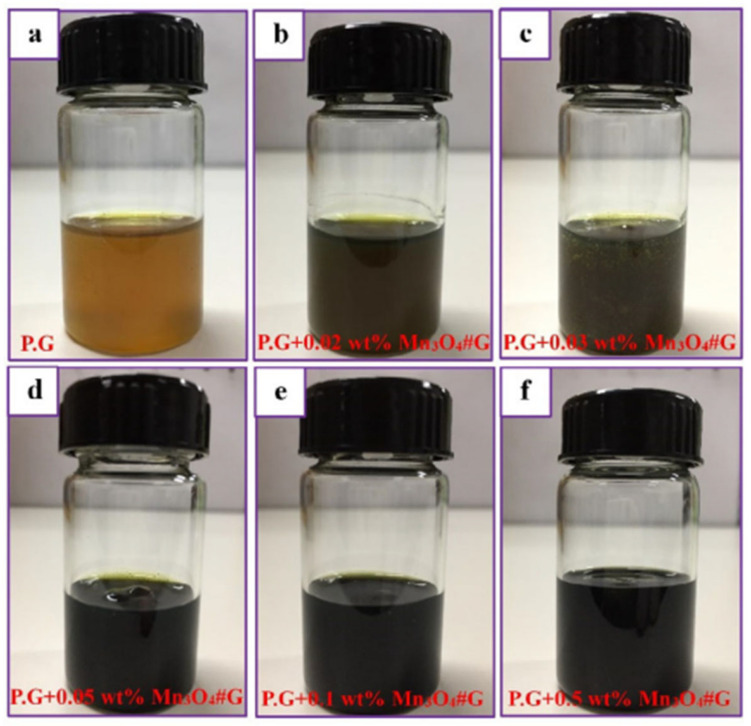
Photographs of lubricating grease samples with different Mn_3_O_4_#G additive contents: (**a**) Base lubricating grease (P.G); (**b**) P.G + 0.02 wt% Mn_3_O_4_#G; (**c**) P.G + 0.03 wt% Mn_3_O_4_#G; (**d**) P.G + 0.05 wt% Mn_3_O_4_#G; (**e**) P.G + 0.1 wt% Mn_3_O_4_#G; (**f**) P.G + 0.5 wt% Mn_3_O_4_#G [30].

Compared with the physical preparation method chemical synthesis is highly accurate and the size, morphology and composition of the nanomaterials can be regulated by precisely controlling the reaction conditions (e.g., temperature, pressure, concentration, etc.). Excellent material properties enable the synthesis of nanomaterials with high purity, homogeneous structure and specific functionality for a wide range of applications [28]. In the chemical preparation process, in situ gelation technology has attracted much attention, whereby gels are formed directly in the chemical reaction system, allowing nanoparticles to be uniformly embedded in the gel network. Wang et al. [29] synthesized uniformly sized nickel nanoparticles using a direct reduction method. After mechanical stirring, they were added to a lithium-based gel-like lubricant. The results showed that when the mass fraction of nickel nanoparticles was 0.2%, the anti-wear and friction-reducing performance of the grease was significantly improved. Jin et al. [30] investigated nanocomposite additives to improve the lubricating properties of grease and synthesized Mn_3_O_4_/graphene nanocomposites as lubricant additives by hydrothermal method. As shown in Figure 2.

Li et al. [31] synthesized a novel thiophosphate, 3-(O,O-dodecylphenol dithiophosphate)-2-methylpropanoic acid (NDMA), and added it as an extreme pressure and anti-wear additive to lithium composite gel-like lubricant. The results showed that NDMA had better shear stability, reduced friction, anti-wear properties, and load-bearing capacity than zinc dialkyldithiophosphate (ZDDP). Wu et al. [32] investigated the tribological properties of chemical composites and physical mixtures of ZnO and SiO_2_ nanoparticles as additives in gel-like lubricants. They selected harder SiO_2_ nanoparticles and softer ZnO nanoparticles, synthesized hard-shell soft-core composite nanoparticles (ZnO@SiO_2_) via chemical vapor deposition, and used them for the first time as lubricant additives. The results showed that the composite ZnO@SiO_2_ nanoparticles exhibited better dispersion and tribological properties than the physically mixed nanoparticles (ZnO/SiO_2_). Yang et al. [33] synthesized titanium dioxide-silica composite nanoparticles (TiO_2_@SiO_2_) by chemical deposition method and doped them into polytetrafluoroethylene (PTFE) grease as additives. The results showed that TiO_2_@SiO_2_ significantly enhanced the friction reduction characteristics and insulating properties of PTFE grease through the surface coating and synergistic effects, demonstrating the potential application value in the field of multifunctional lubricating materials such as high insulating working conditions. Wang et al. [34] prepared calcium fluoride (CaF_2_) nanocrystals with an average particle size of 60 nm using a precipitation method. After friction testing, a composite boundary film composed of CaF_2_, CaO, iron oxide, and organic compounds formed on the friction surface, with a thickness of approximately 12 nm. This effectively reduced friction and wear, providing new design ideas for the development of high-performance nano-additives in gel-like lubricants. Nassef et al. [35] used reduced graphene oxide (rGO) and zinc oxide (ZnO) nanoadditives to modify palm oil grease. ZnO forms a protective zinc compound layer on the metal surface through chemical reactions, achieving boundary lubrication; rGO reduces friction through a unique physical lubrication mechanism, specifically manifested as layer slip effects and surface coverage effects. The two-dimensional layers of rGO form a slip layer between friction surfaces, akin to “molecular-scale sliding plates”, effectively reducing shear resistance. Simultaneously, rGO adsorbs onto the metal surface, forming a physical barrier layer that reduces direct wear through a nano-repair mechanism. Experimental results show that after adding ZnO and rGO, the load-carrying capacity of palm oil grease increases by 30% and 60%, respectively, while the friction coefficient decreases by 60%. This indicates that ZnO and rGO, as nano-additives, significantly enhance the performance of palm oil grease, with rGO exhibiting a more pronounced synergistic effect. Their distinct mechanisms of action complement each other, collectively improving the overall performance of the grease.

Wu et al. [36] synthesized four types of modified carbon quantum dots (CQDs) with different alkyl chain lengths using a hydrothermal method and combined them with lithium-based gel-like lubricants. The study found that the alkyl chain length affects the interfacial adsorption of CQDs and the formation of lubricating films, thereby regulating tribological properties. Longer alkyl chains may enhance interfacial stability and anti-friction effects.

In summary, in the field of preparing gel-like lubricating materials and their composite systems, physical and chemical preparation methods exhibit significant technical differences, as shown in Table 2. Physical preparation techniques, such as mechanical stirring and ultrasonic dispersion, are characterized by their simple operating procedures and low process implementation thresholds, enabling efficient mixing and molding of nanoparticles with gel matrices, and are particularly suitable for large-scale industrial production scenarios. Chemical preparation methods, including sol-gel and emulsion polymerization, can precisely shape the microstructure of gel-like lubricants and the size and morphology of nano-additives through molecular-level precise control. In actual production practice, the preparation of gel-like lubricants requires a comprehensive balance of key factors such as economic cost, production efficiency, and environmental friendliness. For physical preparation methods, advanced techniques such as microfluidic technology and dynamic shear dispersion can be introduced to effectively improve the dispersion precision of nano-additives in the gel matrix. The development of chemical preparation methods should focus on green improvements, such as adopting water-based reaction systems and recyclable catalysts, to reduce production costs and environmental impact. By promoting the deep integration of physical and chemical preparation technologies and continuous process innovation, it is possible to tailor optimal preparation schemes based on different application requirements, thereby maximizing the performance potential of nano-additives in gel-like lubricants.

## 3. Classification of Nano-Additives in Gel-like Lubricants

### 3.1. Metal-Based Nano-Additives

The origin of metal-based nano-additives can be traced back to the early development of nanotechnology [44]. The concept of nanotechnology was first introduced by scientist Taniguchi in 1974 to describe fine machining [45]. However, research on nanoparticles can be traced back much earlier to the 19th century. In 1857, Michael Faraday successfully prepared gold nanosols, one of the first metallic nanoparticles [46]. With the advancement of science and technology, methods such as glow discharge and gas-phase evaporation were developed to prepare a wide range of ultrafine particles of metals and oxides [47]. Until the late 1980s and early 1990s, nanotechnology developed rapidly with the invention of microscopic characterization and manipulation techniques such as scanning tunneling microscopy (STM) and atomic force microscopy (AFM), which allowed us to study the properties and applications of nanoparticles in greater depth and promoted the development and application of metal-based nanoadditives [48]. Metal-based nanoadditives mainly include monolithic or alloy materials such as copper (Cu) nanoparticles, silver (Ag) nanoparticles, nickel (Ni) nanoparticles, and aluminum (Al) nanoparticles [49]. The core advantage of such additives lies in their self-healing functionality, primarily through a nano-repair mechanism. During friction, active substances (wear particles, additive reaction products) or the material itself are “crushed” or “welded” onto the worn surface (especially at defects such as micro-cracks and micro-pits) under the high temperature and pressure of friction contact, filling the damaged areas to restore surface smoothness and form a protective film [50], thereby preventing crack propagation and reducing wear rates. Furthermore, mixing metal-based nanoadditives with base oils can significantly improve the performance of gel-like lubricants [51]. For example, chromate nanoparticles can form passivation films on metal surfaces and inhibit corrosion, and zinc oxide nanoparticles can absorb ultraviolet light and retard polymer aging [52].

**Figure 3:**
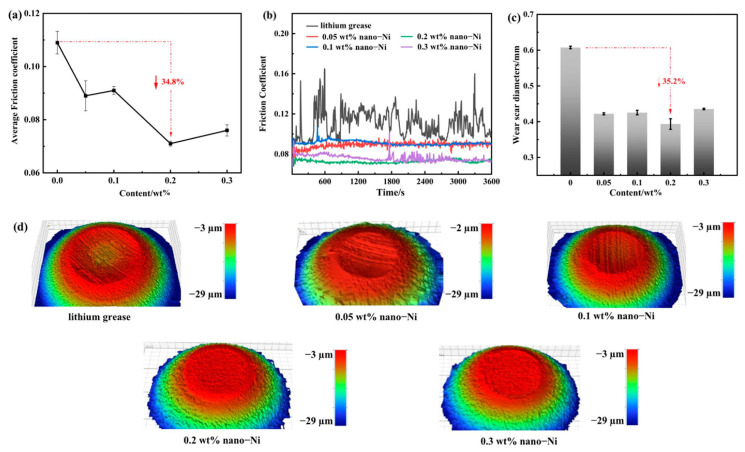
Tribological properties of lithium-based grease and nickel composite materials under point contact mode:(**a**) Average COF; (**b**) COF curves; (**c**) Average WSD; (**d**) WLI morphologies of wear scar [29].

**Figure 4:**
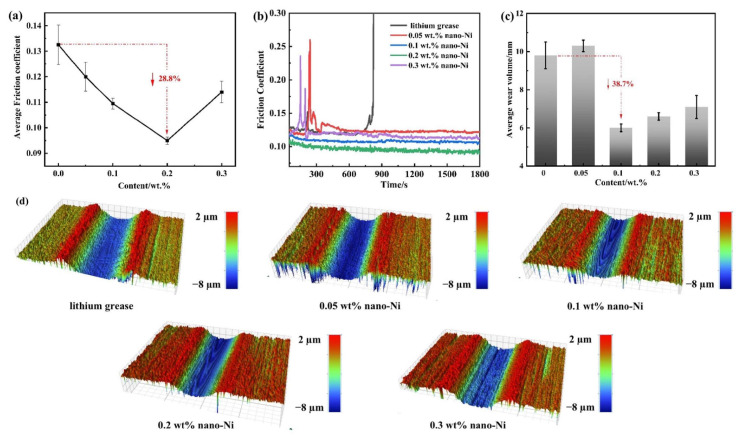
Performance study of lithium-based grease-nickel mixture system in point contact friction: (**a**) Average COF; (**b**) COF curves; (**c**) Average WSD; (**d**) WLI morphologies of wear scar [29].

Wang et al. [29] significantly enhanced the tribological properties of lithium grease by adding 0.2 wt% nickel nanoparticles. The friction reduction and antiwear properties were enhanced by 28.8–34.8% and 35.2–38.7% for point-to-point contact (Figure 3) and point-to-plane contact (Figure 4), respectively. It is shown that nickel nanoparticles are effective additives to improve the performance of lithium grease for complex working conditions and provide a new strategy for the development of high-load, low-wear lubricant materials.

**Figure 5:**
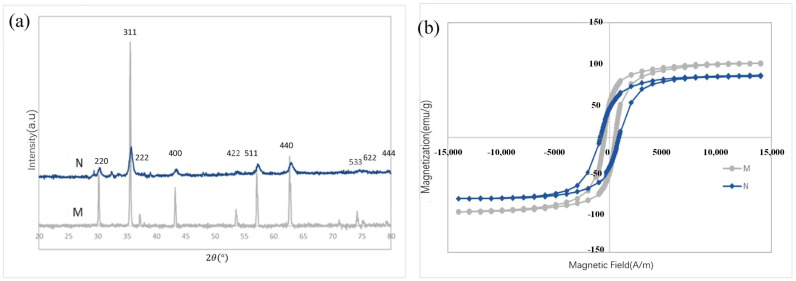
(**a**) XRD diffraction patterns of nano-sized (N) and micron-sized (M) cobalt ferrite, confirming the formation of their crystal structures; (**b**) Magnetic hysteresis loops of the synthesized CoFe2O4 particles that show the magnetization of M is higher as compared to N. (M: CoFe_2_O_4_ particles; N: CoFe_2_O_4_ nanoparticles) [55].

Zhu et al. [53] found that the prepared copper additives can improve the tribological properties and electrical conductivity of lithium-based gel-like lubricants because they can adhere to and deposit on the steel surface that has been subjected to friction, forming a boundary lubrication film composed of cupric oxide, copper oxide, iron oxide, etc. The lubrication film formed can protect the metal from damage. Bai et al. [54] added liquid metal gallium-based liquid metal (GBLM) as an additive to lithium-based gel-like lubricant. Research shows that pure GBLM has poor anti-wear performance under low loads and inadequate lubrication performance under high loads. However, when combined with lithium-based grease, its extreme pressure lubrication capability is significantly enhanced, while its anti-wear performance under low loads remains unaffected. When the mass ratio of GBLM to lithium-based grease is 1:1, the welding load exceeds 10 kN to reach the limit value, indicating that the composite of GBLM and lithium-based grease can enhance extreme pressure performance, maintain low-load anti-wear capability, and exhibit synergistic lubrication potential under extreme high-load conditions. Tarmizi et al. [55] investigated the effect of micro-cobalt and nano-cobalt ferrite (CoFe_2_O_4_) on the viscous behavior of magnetorheological grease. The results show that microcobalt gives the largest reduction in grease viscosity (43.2% reduction), which is superior to nano-CoFe_2_O_4_ (26%), as shown in Figure 5. micrometer-sized particles perform better in terms of viscosity reduction. This suggests that microcobalt has more potential to modulate the rheological properties of magnetorheological materials (e.g., viscosity reduction, flow enhancement). This section focuses on the application of various metal-based nano-additives in gel-like lubricants. Nano-nickel significantly enhances the anti-friction and wear-resistant properties of lithium-based grease (improvement of 28.8–38.7%), making it suitable for complex operating conditions; copper additives improve the tribological properties and conductivity of lithium-based grease by forming a boundary lubrication film; when combined with lithium-based grease, liquid metal GBLM significantly enhances extreme pressure lubrication capability (welding load exceeding 10 kN) while maintaining low-load wear resistance; Micro-cobalt outperforms nano-cobalt ferrite in regulating the viscosity (reduction rate of 43.2%) and flowability of magnetorheological lubricating grease. Through these studies, it can be concluded that different material additives optimize lubricating grease performance through mechanisms such as film protection, synergistic lubrication, or rheological regulation, providing diverse strategies for the development of high-performance lubricating materials under high-load and extreme operating conditions. The unique three-dimensional network structure of hydrogels and organic gels can serve as a dispersion carrier and performance regulation platform for nano-metal particles. The polymer network of the gel restricts the Brownian motion of nanoparticles through physical confinement, effectively inhibiting particle agglomeration and maintaining their nanoscale dispersion state. Additionally, the gel’s swelling properties confer dynamic response capabilities to the lubrication system: under friction heat or pressure, the gel absorbs the surrounding medium (such as water or organic solvents) and swells, prompting the encapsulated nano-metal particles to migrate toward the friction interface, thereby achieving targeted release of the additives. The composite of the gel and nano-metal particles produces a synergistic effect: the gel’s viscoelastic properties cushion the impact on the friction pair, while the nano-particles enhance the load-bearing capacity of the lubricating film. Together, they improve the overall performance of the lubricating material.

### 3.2. Metal Oxide Nanoadditives

**Figure 6:**
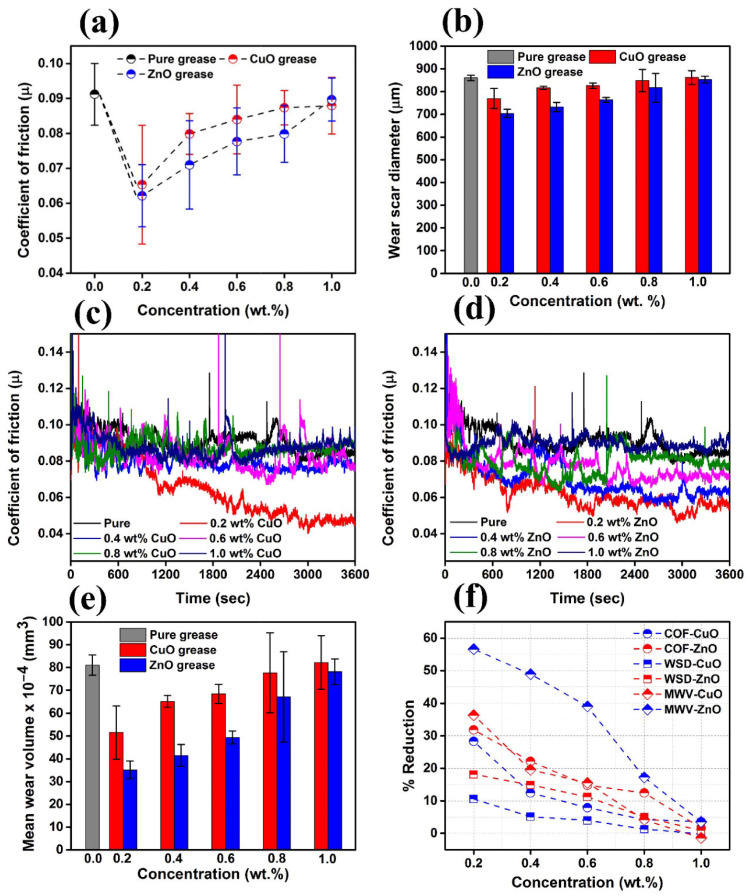
(**a**) Effect of different concentrations of ZnO and CuO nanoparticles on the average coefficient of friction (COF). (**b**) Influence of changes in ZnO and CuO nanoparticle concentrations on the wear scar diameter (WSD). (**c**) Time-dependent characteristics of the coefficient of friction with varying concentrations of CuO nanoparticles. (**d**) Time-dependent characteristics of the coefficient of friction with varying concentrations of ZnO nanoparticles. (**e**) Effect of varying concentrations of ZnO and CuO nanoparticles on the average wear volume (MWV). (**f**) Percentage reduction in COF, WSD, and MWV caused by ZnO and CuO nanoparticles [64].

The origin of metal oxide nano-additives is closely related to the development of nanotechnology [56]. The concept of nanotechnology was first introduced by Richard Feynman in 1959, and with the advancement of science and technology, especially the invention of the microscope, scientists are able to observe and manipulate materials on the nanoscale. Typical materials include titanium dioxide nanoparticles, silicon dioxide nanoparticles, zinc oxide nanoparticles, and alumina nanoparticles, which are characterized by high chemical stability, oxide surface inertness, and resistance to high-temperature oxidation [57]. With high load-bearing capacity, hard oxides (e.g., Al_2_O_3_) disperse the contact stresses through the “micro-support effect” to enhance the extreme pressure performance [58]. He et al. [59] used nano-Al_2_O_3_, nano-ZnO, and composite nano-particles of the two as additives in gel-like lubricants. They found that when the Al_2_O_3_ content in the additives was 0.4 wt% and the ZnO content was 0.6 wt%, the composite nano-particle-based gel-like lubricant had the lowest average COF and wear scar diameter, which were approximately 160% and 28% lower than those of the base lubricant, respectively. Wu et al. [60] prepared three base lubricants—composite gel lithium-based (LCG), polyurea (PG), and composite calcium sulfonate (CSCG)—as well as corresponding lubricants containing 1% mass fraction copper oxide (CuO) nanoparticles (six types in total). The results showed that the polyurea lubricant (PG) containing CuO exhibited the best tribological performance, with significantly reduced friction coefficients and wear scar diameters, and a smoother and finer wear surface. The performance improvement was attributed to the synergistic lubrication mechanism between the layered thickener structure of the polyurea lubricant and the CuO nanoparticles. He et al. [61] found that adding 0.6 wt% of nano-cerium oxide (CeO_2_) significantly improved the friction reduction (friction coefficient reduced by 28%) and anti-wear (wear scar diameter reduced by 13%) properties of lithium-based gel-like lubricants. At 50 °C, the friction coefficient and wear width of the lubricant reached their lowest values, indicating that the performance was optimal at this temperature. Wu et al. [62] used zinc oxide-silicon dioxide core-shell composite nanoparticles (ZnO/SiO_2_) as additives in gel-like lubricants and found that under light loads, the coefficient of friction decreased by 18% and the diameter of the wear scar decreased by 22%. Under heavy loads, the anti-friction and wear-resistant effects were even better, with the coefficient of friction decreasing by 15% and the diameter of the wear scar decreasing by 28%. The synergistic interaction between ZnO and SiO_2_ in the core-shell structure enhances the stability and load-bearing capacity of the interface lubrication film, indicating that these composite particles significantly improve grease performance under different load conditions. Du et al. [63] found that composite nanoparticles containing grease exhibited a low coefficient of friction and excellent anti-wear properties. When the ratio of TiO_2_ to CeO_2_ was 6:4, the samples showed the best tribological properties, with a 30.5% reduction in the coefficient of friction and a 29.2% reduction in the diameter of the wear point compared to the base grease. In addition, the roughness of the wear point surface and the maximum depth of the wear marks were significantly reduced. Sooraj Singh Rawat et al. [64] showed the greatest reduction in mean wear volume (MWV) (~36% and ~56%) by adding CuO and ZnO nano-additives at 0.2% *w*/*w*, respectively, in paraffin grease. The energy consumption was reduced by ~31% and 28% by dispersing ZnO and CuO nano-additives in paraffin grease at the same concentration, as shown in Figure 6.

Qiang et al. [65] studied the performance of nanorods-Al_2_O_3_ as a grease additive and found that it can significantly improve lubrication performance. When the addition amount is 0.3 wt%, the average friction coefficient and scratch diameter of the grease are the lowest, the worn steel surface is smooth with no obvious defects, and there is a correlation between the tribological and rheological properties of lithium grease. Additionally, the composite of nano-metal oxides (such as Al_2_O_3_, TiO_2_) with gel materials is an important direction in the field of lubrication. Among these, nano-Al_2_O_3_, due to its high hardness, chemical stability, and good dispersibility, can fill micro-pits on friction surfaces and enhance the load-bearing capacity of the lubricating film. Titanium dioxide (TiO_2_) exhibits outstanding performance during friction processes due to its unique photocatalytic and self-healing properties. The mechanisms of its photocatalytic and self-healing effects are as follows: Under friction heat or light exposure, TiO_2_ may promote the decomposition of surface oxides through photocatalytic effects or undergo chemical reactions with metal surfaces to form low-shear-strength titanium compounds, such as titanates formed by TiO_2_ and metals. These compounds fill surface defects and form a lubricating film. The protective film has low shear strength, enabling interlayer slippage during friction to reduce friction resistance while preventing wear caused by abrasion from hard particles. Additionally, the introduction of smart gel materials (such as thermosensitive, pressure-sensitive, or pH-responsive polymer gels) provides new avenues for precise regulation of nanoadditives, potentially further optimizing the performance of TiO_2_ and other nanoadditives in lubrication applications. This section systematically explores the application mechanisms and performance optimization pathways of metal oxide nanoadditives in gel-like lubricants. The results indicate that different types of additives, through diverse mechanisms such as interfacial film formation, synergistic lubrication, and functional response, provide a systematic solution for the development of high-performance lubricants under extreme operating conditions. The introduction of smart gel materials (such as thermosensitive, pressure-sensitive, or pH-responsive polymer gels) has provided new avenues for precise control of nano-additives. As carriers, smart gels can dynamically regulate the release and distribution of nano-oxides through physical or chemical response mechanisms in response to changes in environmental temperature or pressure. The “smart carrier-nano-additive” synergistic system not only achieves dynamic optimization of lubrication performance but also endows lubrication materials with self-regulatory capabilities, providing innovative strategies for friction control under extreme conditions (such as high temperatures, heavy loads, and complex environments).

### 3.3. Carbon Nanomaterial Additives

**Figure 7:**
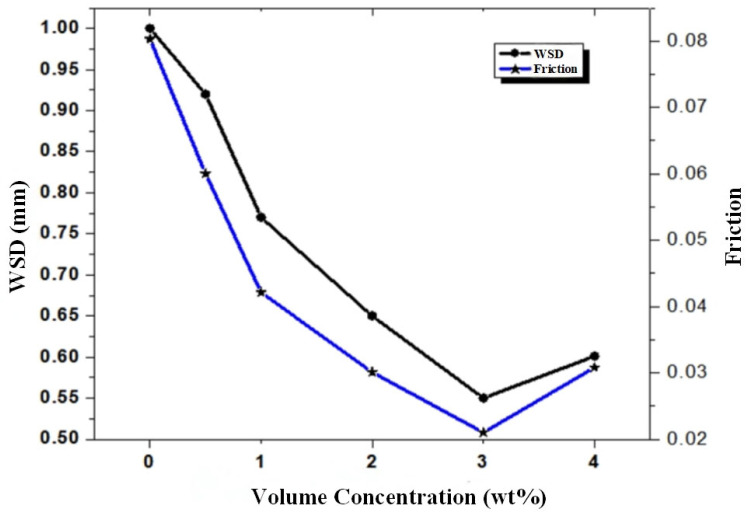
Functional relationship between graphene concentration gradient and friction coefficient. (Reprinted with permission from Reference [21]. Copyright 2016 Engineering & Technology).

The origin of additives for carbon nanomaterials can be traced back to the late 1980s and early 1990s, and has evolved with the discovery of carbon nanomaterials and further research [66]. Buckminsterfullerene C_60_ was first reported in 1985 by Kroto et al. [67]. Fullerene is a spherical molecule composed of 60 carbon atoms through 12 five-membered and 20 six-membered rings, and is considered to represent a zero-dimensional nanocarbon material due to its unique structure and properties [68]. Carbon nanotubes were discovered in 1991 by scientist Iijima [69]. Carbon nanotubes are circular tubular structures formed by hybridization of carbon atoms in SP_2_ and SP_3_, which have excellent electrical conductivity and structural buildability [70]. The discovery of carbon nanotubes has further promoted the study of carbon nanomaterials. In 2004, AndreGeim and KonstantinNovoselov succeeded in isolating a single layer of graphene from graphite [71]. Graphene is a two-dimensional material composed of a single layer of carbon atoms with extremely high electrical and thermal conductivity and mechanical strength [72]. Its unique advantage lies in the laminar lubrication mechanism, where graphene and C_60_ reduce the coefficient of friction by interlayer slip [73]. With the continuous progress of the preparation technology of nanocarbon materials and the in-depth study of their properties, the application fields of nanocarbon material additives are gradually expanding [74]. Nanocarbon materials such as nanodiamond and graphene are used to prepare high-performance grease additives [75]. Rukhov et al. [76] investigated the performance impact of graphite nanoplatelets (GNP) as an additive in gel-like lubricants. As the GNP concentration increased from 0 to 150 ppm, the residual water content of the lubricant rose from 0.6 wt% to 2.3 wt%, while penetration decreased from 44 mm to 31 mm (increased consistency). At a GNP concentration of 150 ppm, the wear spot size was smallest (0.215 mm), and the anti-wear performance was optimal. A balance must be struck between optimizing performance and controlling moisture content when determining the application concentration of GNP. Kamel et al. [21] found that graphene nanosheets (GNS) significantly improve the performance of calcium-based gel-like lubricants when used as additives: friction was reduced by 61%, the wear scar diameter (WSD) was reduced by 45%, and the anti-wear performance was significantly enhanced; at the addition of 3 wt% of GNS, the extreme pressure performance (EP) was enhanced by 60%, showing excellent high load stability. This indicates that GNS can significantly improve the comprehensive performance of the grease, especially in the friction reduction, anti-wear and extreme pressure, as shown in Figure 7.

**Figure 8:**
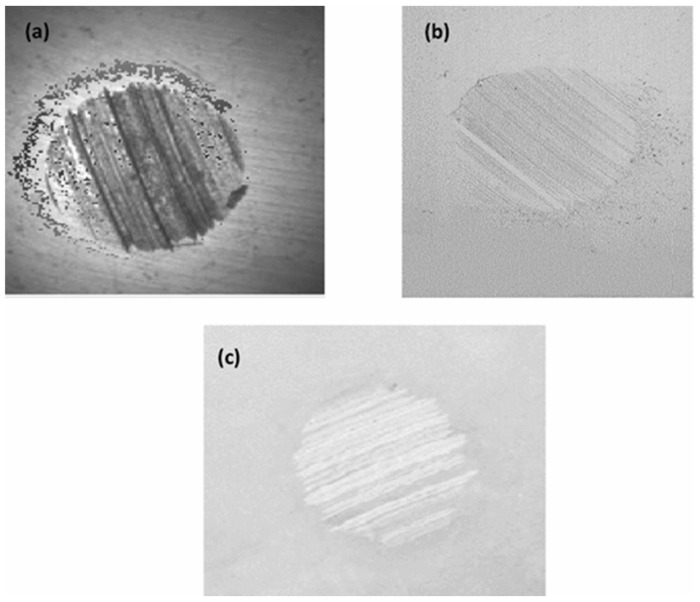
(**a**) Basic lithium-based composite grease (LCG). (**b**) LCG/MWCNTs containing 4 wt% multi-walled carbon nanotubes. (**c**) LCG/Al_2_O_3_ containing 4 wt% aluminum oxide [78].

Kumar et al. [77] investigated the effect of different particle sizes of graphite nano-additives on tribological properties and showed that all sizes were advantageous for all selected properties and the larger the particle size, the lower the performance improvement. Kamel et al. [78] found that adding multi-walled carbon nanotubes (MWCNT) and aluminum oxide (Al_2_O_3_) at a ratio of 4 wt% to a lithium-calcium-based gel-like lubricant significantly optimizes its performance. At this point, the grease exhibits the lowest wear scar diameter (WSD) and friction coefficient, with optimal anti-wear and friction-reducing properties. Additionally, the weld point increases by 26%, the drop point by 32%, and the thermal conductivity by 75%, demonstrating excellent extreme pressure performance, high-temperature stability, and thermal conductivity, as shown in Figure 8.

**Figure 9:**
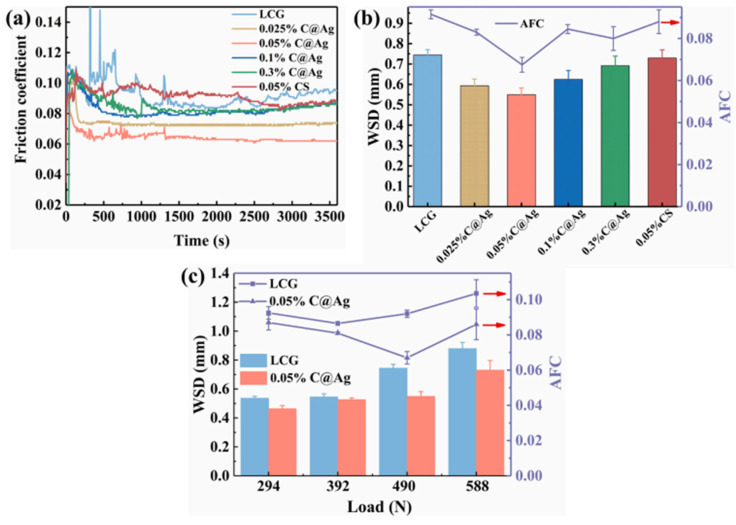
Friction coefficients (**a**) and corresponding AFC and WSD results (**b**,**c**) for various grease samples. (Reprinted with permission from Reference [80]. Copyright 2024 Elsevier).

Goti et al. [79] utilized innovative nano-additives such as graphene to improve the performance of conventional lubricants and showed that the right amount of graphene reduces friction and wear because graphene enhances the resistance of the lubrication film to wear. However, excessive amounts are harmful and interfere with lubrication. Ren et al. [80] found that C@Ag core-shell materials, when used as gel-like lubricant additives, can reduce the average friction coefficient of grease by 27.17% and decrease the diameter of wear spots by 26.12%. The mechanism behind this enhanced performance lies in the hard carbon core (C) providing support and wear resistance, while the soft silver shell (Ag) performs lubrication and self-repair functions. The two work synergistically to achieve rolling, filling of wear pits, and surface self-repair effects, significantly enhancing the lubricant’s overall performance, as shown in Figure 9.

**Figure 10:**
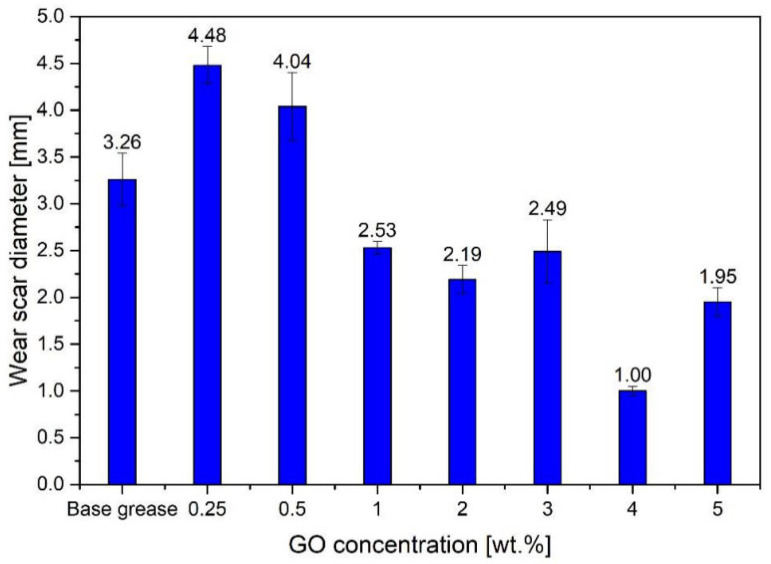
The Impact of GO Additive Content on the Tribological Properties of Lubricants [83].

**Figure 11:**
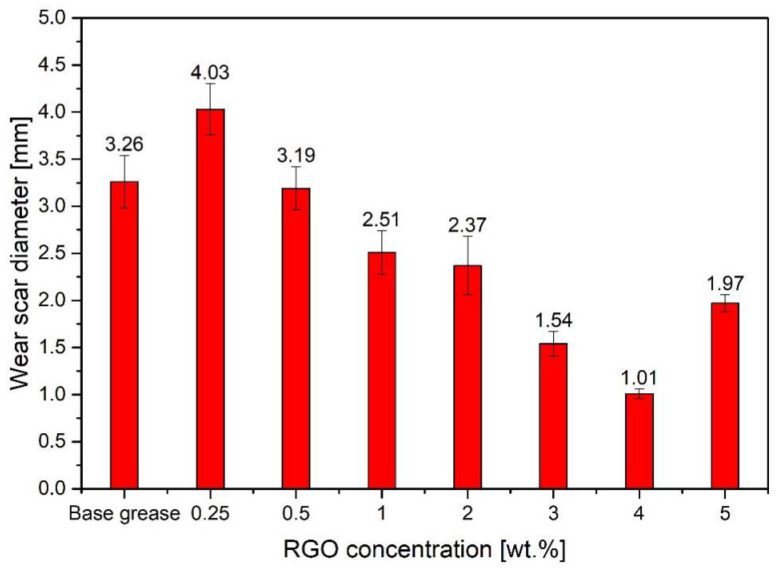
The Impact of RGO Additive Content on the Tribological Properties of Lubricants [83].

Li et al. [81] studied the tribological properties of gel-like lithium-based grease with a small amount of graphene (FLG) added. The results showed that when the mass fraction of few-layer graphene (FLG) was 0.1%, the reduction rate of the coefficient of friction was the highest, and the wear scar diameter (WSD) was the smallest. Wang et al. [82] used AI-assisted design to develop chemically functionalized graphene (FGR) and functionalized carbon nanotubes (FCNT) as grease additives. When the FGR content was 0.14 wt% and the FCNT content was 0.16 wt%, the friction-reducing performance of the grease improved by 25.66%, and the anti-wear performance improved by 29.34%. The performance optimization stems from the synergistic lubricating effect of FGR and FCNT. This study utilized AI to optimize the functionalization process, achieving efficient lubrication at extremely low addition levels while maintaining cost-effectiveness. Djas et al. [83] used flake graphene (graphene oxide GO and reduced graphene oxide RGO) as grease additives and found that it exhibited excellent antiwear properties at a concentration of 4 wt%. Compared with the base lubricant, the wear scar diameter (WSD) was reduced by nearly 70%, which significantly reduces wear and improves lubricant durability, as shown in Figure 10 and Figure 11. Graphene exhibits an extremely strong anti-wear effect at high concentrations, and it is suitable for high load and high wear conditions.

Sun et al. [84] studied the effect of graphene and graphite composite additives on the micro-motor tribological behavior of steel wires in grease. The results show that the addition of multilayer graphene or micron-sized graphite can significantly improve the anti-wear ability of the grease, and the composite additive makes the micro-cutting phenomenon on the wear surface significantly reduced, effectively inhibiting the material surface damage. The synergistic effect of graphene and graphite optimizes the tribological performance of the grease, which is especially suitable for precision or high wear scenarios such as steel wire microfriction. From the perspective of manufacturing industry, nanocarbon material additives can significantly improve the performance of materials, in metal processing, it can improve the strength and toughness of the metal, so that the metal products produced are more durable, which is conducive to improving product quality, and then improve production efficiency and reduce production costs. In chemical production, it helps to optimize the reaction process, enhance the activity of the catalyst, making chemical production more efficient and environmentally friendly. Not only that, nanocarbon additives in the biological field also has important applications. Xie et al. [85] used multilayer graphene additives to enhance the lubricity of bio-lubricants. The results indicated that the addition of multilayer graphene can improve the load-carrying capacity and high-temperature lubricity of natural wax. This section focuses on the application of nanocarbon material additives in gel-like lubricants, finding that carbon-based materials such as graphite nanoplatelets, graphene, and carbon nanotubes, as well as aluminum oxide, can significantly enhance the lubricant’s anti-friction, wear resistance, extreme pressure, and high-temperature stability through mechanisms such as film formation, filling, and synergistic lubrication. Additive concentration and particle size have a significant impact on performance. AI-assisted design and composite additives (such as graphene and graphite) can optimize lubrication performance, providing direction for the development of high-performance lubrication materials.

In summary, the addition of carbon nanomaterials can significantly enhance the load-bearing and anti-wear capabilities of lubricating grease, driving the development of gel-based lubricating grease technology. However, their large specific surface area and high surface energy make them prone to agglomeration, leading to uneven dispersion in lubricating grease. In the future, nanocarbon materials will develop in the direction of green and low-cost applications. Systems formed by the composite of carbon-based nanomaterials (such as carbon nanotubes and graphene) with polymer gel matrices hold great potential in the field of tribology. The unique viscoelastic properties of this composite gel system allow carbon-based nanomaterials to distribute uniformly across the contact interface during friction, enabling adaptive deformation to reconstruct the surface. Under shear stress, nanocarbon materials align in an oriented manner, while the reversible deformation of the gel phase provides movement space, enabling them to fill microscopic defects in the friction pair, reduce surface roughness, and lower the friction coefficient. Additionally, the high mechanical strength and low shear resistance of carbon-based nanomaterials, combined with the viscoelastic properties of the gel phase, form an efficient energy dissipation system, offering new insights for the development of high-performance gel-like lubricants.

### 3.4. Other Nano Additives

Other types of nano-additives are used in a variety of applications and can be applied to industrial fields, plastic modification and so on. Nucleus-shell structured additives are a novel class of materials that achieve functional synergy through a hierarchical “core-shell” design. Their unique structural advantages confer irreplaceable application value across multiple industrial and research fields. The core primarily provides foundational properties such as mechanical support, catalytic activity, and magnetic response. The shell, on the other hand, primarily regulates these functions by imparting additional properties such as dispersibility, compatibility, and responsiveness through surface modification and interface modification techniques. Interface synergy is achieved through chemical bonding or gradient transition layers between the core and shell, which suppress phase separation and enable complementary performance. Wang et al. [86] used vacuum impregnation to adsorb ionic liquids (ILs) onto PTFE@dendritic silica (DFNS) core-shell particles, successfully preparing soft/hard/liquid three-phase composite particles (PTFE@DFNS@ILs). Adding these particles to epoxy resin coatings significantly improved the coatings’ hardness, modulus, and anti-friction wear resistance. The superior performance stems from the synergistic effects of PTFE’s lubrication, DFNS’s rolling wear resistance, and ILs’ promotion of transfer film formation. This work provides a new method for introducing liquid lubricants into micro/nano composite particles, demonstrating the immense potential of solid-liquid-core-shell structures in the field of lubrication. Liu et al. [87] developed a multi-layered core-shell structured nanocomposite C@SiO_2_@C using polymethyl methacrylate (PMMA) and ethyl cellulose as carbon sources to replace traditional sulfur/phosphorus-containing friction modifiers with stronger corrosive properties. When 4.0 wt% of this additive was added to PAO10 base oil, the friction coefficient and wear volume were significantly reduced by 45.8% and 75.6%, respectively. Its excellent lubricating performance is attributed to a triple-action mechanism: the high load-bearing capacity of spherical particles disperses contact stress, the carbon components released upon particle fracture form a physical isolation deposit film at the friction interface, and the friction-induced chemical reactions at the interface generate a protective friction chemical film. This dual synergistic mechanism of physical barrier and chemical film formation makes C@SiO_2_@C nanocomposites a promising candidate additive for industrial applications, combining low corrosion and high-efficiency lubrication performance. Haque et al. [88] employed a simple hot water treatment process (75 °C constant temperature for 24 h) to directly grow three-dimensional leaf-like CuO nanostructures on the surface of copper powder, successfully preparing a binder-free core-shell type CuO/Cu electrode. The microstructure, elemental composition, crystal structure, and high specific surface area characteristics of the electrode were confirmed through multi-dimensional characterization methods. Electrochemical testing showed that in Na_2_SO_4_ electrolyte, the electrode exhibited a specific capacitance of approximately 220 F/g at a scan rate of 5 mV/s, although the capacitance retention rate was approximately 38% after 1500 cycles. The research results indicate that this CuO/Cu core-shell material prepared via hot water treatment technology demonstrates excellent application potential as a pseudocapacitive supercapacitor electrode, and its simple synthesis process provides new insights for innovations in energy storage technology. Incorporating nano additives into plastics, such as nano calcium carbonate and nano montmorillonite, can improve the mechanical properties of plastics, increase the strength, hardness, toughness, etc., and also enhance the heat resistance, water resistance, and barrier properties of plastics [89]. Surface-grafted nanoparticles refer to a class of composite materials in which functional molecular chains (such as polymers, organic small molecules, biomolecules, etc.) are introduced onto the surface of nanoparticles via chemical or physical methods. The primary targets for grafting are active sites on the surface of nanoparticles (such as SiO_2_, TiO_2_, Fe_3_O_4_, graphene, etc.), such as hydroxyl groups and unsaturated bonds, which form covalent bonds or physical adsorption with functional molecules. Grafting can improve the dispersion of nanoparticles in solvents/matrices, enhance interface compatibility with polymer matrices, and confer specific functionalities (such as magnetic properties, catalytic activity, and bio-targeting). Haque et al. [90] proposed a two-step modification process (methacrylic acid graft polymerization + in situ introduction of zinc oxide nanoparticles), successfully preparing ZnO NPs-modified polyamide composite reverse osmosis membranes (ZnO NPs-PMAA-g-PA (TFC)). Characterization results indicated that ZnO nanoparticles were stably anchored on the membrane surface. The modified membrane material exhibits significantly enhanced hydrophilicity (water contact angle reduced to ~50°), along with excellent desalination performance: total salt rejection rate of 97%, with 99% retention of divalent ions and 98% retention of monovalent ions, and potential resistance to Escherichia coli contamination. This modification method, by synergistically enhancing the membrane material’s hydrophilicity, selective permeability, and resistance to biological contamination, provides an effective solution for optimizing the performance of reverse osmosis membranes used in seawater desalination. Lin et al. [91] revealed the critical importance of maintaining the highly dispersed state of nanoparticles (NPs) throughout the entire process of preparing polymer-grafted nanoparticle (PGNP) gas separation membranes. Studies have shown that even if the initial NPs exhibit good dispersion, surface functionalization treatment can still cause NP agglomeration (equivalent to an increase in core particle size), leading to a significant decline in the permeability performance of the final PGNP membrane. By introducing a protective layer before functionalization to inhibit NP agglomeration and then implementing grafting modification, PGNP membrane materials with significantly improved permeability performance were successfully prepared. The core mechanism lies in the fact that, under the same grafting parameters, nanoscale-dispersed NPs exhibit superior membrane separation performance compared to agglomerated NPs. This result highlights that strictly controlling the dispersion state of particles throughout the preparation process is a core technical element for optimizing the separation performance of PGNP membranes. Nayak et al. [92] used GISAXS and XRR characterization techniques to discover that polyethylene glycol (PEG)-grafted silver nanoparticles (AgNPs), gold nanoparticles (AuNPs), and their mixed systems can self-assemble at the gas-liquid interface to form environmentally stable hexagonal ordered thin film structures. Among these, nanoparticles grafted with short-chain PEG exhibit a dense, ordered arrangement, while those with long-chain PEG form a low-density quasi-bilayer structure. Due to their higher grafting density, AuNPs exhibit significantly superior ordered arrangement at the interface compared to AgNPs. In the binary mixed system, AuNPs dominate the gas-liquid interface, while AgNPs embedded in the system cause lattice parameter expansion, but no formation of a binary superlattice structure was observed. This study provides an important theoretical foundation for understanding the interface assembly mechanism of PEG-grafted nanoparticles and optimizing the preparation of ordered films.

In the field of biomedicine, gel-like lubricants have emerged as highly promising multifunctional biological carriers due to their unique rheological properties and biocompatibility. Their continuous-phase network structure enables the formation of stable composite systems with nanomaterials, thereby enhancing the dispersion and stability of nanocarriers within biological systems. Common drug carriers such as nanoliposomes and nanopolymers, though possessing good biocompatibility and modifiability, tend to aggregate or undergo rapid metabolism in vivo, affecting drug efficacy. However, dispersing them in gel-like lubricants can utilize the gel’s three-dimensional network to physically confine nanoparticles, slowing degradation rates and enhancing drug controlled-release performance [93]. Cheng et al. [94] addressed the challenge of tissue adhesion commonly encountered during Achilles tendon repair by developing an injectable lubricating hydrogel (ILH) with motion responsiveness and anti-adhesion properties. In vitro cell experiments and coagulation tests demonstrated that ILH exhibits excellent cell compatibility and blood compatibility. In functional validation, it significantly prevents tendon adhesion and promotes tendon healing, providing an innovative material solution for addressing adhesion issues in Achilles tendon repair.

The swelling properties of the gel enable it to achieve controlled release of the nanomedicine carrier in response to changes in the local microenvironment (such as pH, temperature, and enzyme concentration), thereby further enhancing the efficiency and precision of drug targeted delivery. This gel-nanomaterial composite system not only reduces the toxic side effects of drugs on normal tissues but also provides innovative solutions for biomedical applications such as cancer treatment and tissue repair [95]. In the field of environmental protection can be carried out in the treatment of wastewater, nano titanium dioxide, nano activated carbon, etc. has a large specific surface area and adsorption properties, can be used in wastewater treatment, can adsorb and degrade heavy metal ions, organic matter and other pollutants in the water, to achieve the purpose of purifying the water quality [96]. Wang et al. [34] found that CaF_2_ nanocrystals as grease additives have excellent antiwear, friction reduction and extreme pressure properties. The additive amount was not linearly positively correlated with the grease performance, and an optimal additive ratio existed. CaF_2_ nanocrystals can significantly improve the comprehensive tribological properties of grease, but the additive amount needs to be optimized to achieve the best effect. Senyk et al. [97] found that additives with smaller particle sizes, a larger proportion of the nanoparticle fraction, and a more developed porous structure were more effective by investigating the effects of nano-hexagonal boron nitride and micrometer particles on the structure, thermal and mechanical stability of lithium-calcium based greases. Wang et al. [98] studied polyurea grease containing molybdenum dialkyl dithiophosphate (MoDDP) and potassium borate (PB) additives. The results showed that MoDDP, as a polar additive, significantly enhanced the colloidal stability of the grease, increased its apparent viscosity, and improved its structural strength; however, PB had a minimal effect on the colloidal structure and structural strength of the grease. This indicates that different additives have notable discrepancies in their modulatory effects on grease performance. Shu et al. [99] compared the performance of polypropylene (PP) thickened grease and lithium complex (Li-composite) grease to test the effect of pure grease and the addition of 2 wt% ZDDP and/or MoDTC. It was found that in polypropylene greases, the combination of ZDDP and MoDTC showed optimal performance with the lowest coefficient of friction and longer service life, and demonstrated broad applicability independent of sliding speed, contact pressure, temperature or type of sliding. Polypropylene-based greases offer a better overall performance than lithium complex greases with the same additive formulation. The synergistic action of the polypropylene thickener and the ZDDP+MoDTC additive package significantly improves the friction reduction and durability of the grease for a wide range of operating conditions. Kumar et al. [100] investigated the performance characteristics of lithium grease containing PTFE particles as additives, lithium based synthetic grease with polyalphaolefin as base stock and different sizes of PTFE particles (4 wt%), 50 nm, 6 µm, 9 µm and 12 µm and shape as functional additives. The results show that the smaller the size, the better the overall performance. Zhong et al. [101] used a variety of analytical methods to explore the effect of temperature on the characteristics of baijiu. The results showed that temperature can significantly alter the content of volatile components, ethanol-water structure, intermolecular forces, and viscosity of baijiu, thereby affecting its mouthfeel and flavor perception. The study further clarified that baijiu exhibits optimal mouthfeel and flavor experience at 25 °C, providing a scientific basis for selecting the optimal drinking temperature for baijiu.

Yan et al. [102] investigated the effect of calcium borate as an additive on the tribological properties of gel-based lithium-based grease and polyurea-based grease. The results showed that calcium borate forms a boundary lubrication layer with the soap fibers in the grease, significantly optimizing the tribological properties. SEM analysis revealed that the soap fibers in polyurea-based grease were more dispersed, smaller, and shorter, while those in lithium-based grease had a more compact structure. The polyurea-based grease exhibited superior anti-wear and friction-reducing properties due to its more dispersed soap fiber structure and higher oxide content. Nano-additives, with their unique physicochemical properties, have demonstrated outstanding performance and broad application potential across various fields such as electronics, catalysis, and biomedicine. The viscoelasticity of the gel synergizes with the high strength and high modulus characteristics of nano-additives, enabling the composite grease to maintain excellent lubrication performance under extreme conditions such as heavy loads and high temperatures. In summary, the composite of nano-additives and gel-based grease not only optimizes and upgrades the original performance of grease but also provides innovative solutions for high-end equipment lubrication, demonstrating broad application prospects. This section systematically explores the applications and innovations of nano-additives in the fields of biomedicine, environmental protection, and lubricant materials. In the field of biomedicine, the gel swelling properties of nano-additives enable nano-drug carriers to achieve microenvironment-responsive release, thereby enhancing the precision of targeted therapy; In environmental protection, nanomaterials with high adsorption capacity effectively treat pollutants in wastewater. In lubrication materials, additives such as CaF_2_ and hexagonal boron nitride enhance the anti-wear and friction-reducing properties of lubricants through optimized ratios, particle sizes, and synergistic interactions with the matrix. New systems based on polypropylene demonstrate superior adaptability to various operating conditions, providing critical support for technological breakthroughs across multiple fields. Table 3 summarizes the different types of additives and their application areas.

## 4. Performance Analysis and Evaluation of Nano Additives in Gel-like Lubricants

### 4.1. Anti-Wear

**Figure 12:**
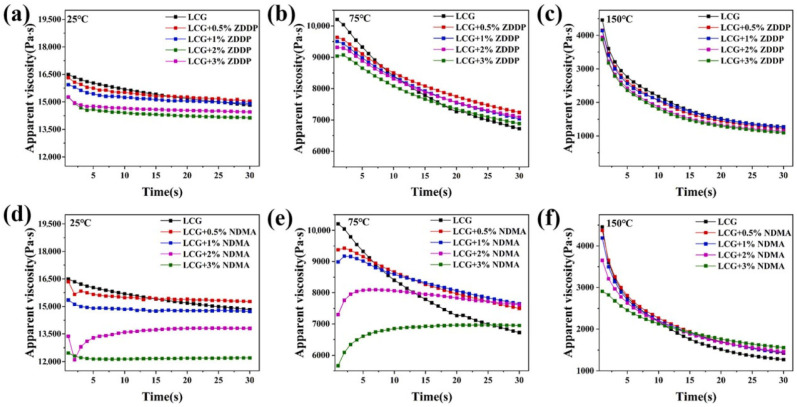
Apparent viscosity curves of LCG containing ZDDP (**a**–**c**) and LCG containing NDMA (**d**–**f**) at 25 °C, 75 °C, and 150 °C [31].

Wear refers to the surface of the object in relative motion, due to mechanical, chemical and other factors that lead to the gradual loss of material or surface properties of the phenomenon of change, will cause harm to equipment, production, safety and other aspects. Mechanical equipment can reduce accuracy, such as machine tools and instrumentation can lead to increased fit clearance, decreased motion accuracy, increased energy consumption, increased surface roughness of parts, and increased frictional resistance [106]. In the mechanical transmission process, in order to overcome the increased friction, the equipment needs to consume more energy to maintain operation, resulting in lower energy utilization and increased energy consumption [107]. Excessive wear and tear on critical components may lead to sudden equipment failure downtime [108]. It can produce very serious hazards in the production process, affecting the efficiency in industrial production, leading to a decline in equipment performance, unstable operation, and the need for frequent downtime for maintenance and replacement of parts [109]. Wear and tear not only affects the accuracy of the equipment itself, but also directly affects the quality of the product and cause serious safety accidents [110]. Gel-like lubricant nano-additives play a crucial role in preventing wear and tear [111]. Extreme pressure antiwear additives are mainly suitable for extreme working conditions such as high loads and high temperatures, where the contact pressure on the surfaces of mechanical parts is extremely high, and the oil film formed by ordinary grease is extremely easy to be destroyed [112]. Extreme pressure antiwear additives will play a key role at this point, and they mainly contain active elements such as sulfur, phosphorus, and chlorine [113]. When the contact pressure on the component surfaces reaches a certain level, these additives will react chemically with the metal surfaces to generate a chemically reactive film with low shear strength, which effectively separates the metal surfaces and prevents them from coming into direct contact with each other under high loads, thus greatly reducing the coefficient of friction and minimizing wear [114]. Li et al. [31] applied the novel thiophosphate 3-(O,O-dinonylphenol dithiophosphate)-2-methylpropionic acid (NDMA) as an extreme pressure (EP) and antiwear additive in lithium complex grease. It was found that NDMA significantly enhanced the oil film stability and improved the oil separation performance of the grease under high pressure and high temperature conditions. Compared with conventional ZDDP additives, NDMA exhibits superior shear resistance, better friction reduction, stronger wear resistance and higher load carrying capacity, as shown in Figure 12.

Shen et al. [115] conducted a systematic evaluation of the tribological performance of lithium-based grease (LG) under the influence of three single additives (potassium borate PB, zinc dialkyldithiophosphate ZDDP, molybdenum dialkyldithiophosphate MoDDP) and two composite additives. Among these, MoDDP undergoes thermal decomposition during friction, releasing S and Mo elements that react with the metal surface to form nanoscale MoS_2_ layers. The interlayer van der Waals forces of these layers are weak, facilitating slip, while also filling surface grooves to form a “repair-type” lubricating film. Meanwhile, PB hydrolyzes or oxidizes to form borate/oxides, forming a hard and dense protective layer on the metal surface. This layer complements the soft MoS_2_ layer, creating a synergistic effect that enhances the film’s load-bearing capacity while reducing surface defects through “repair,” thereby lowering wear. The results indicate that the PB+MoDDP composite additive exhibits the best overall performance, significantly improving friction-reducing performance, enhancing wear resistance, and enhancing extreme pressure (EP) performance. Due to the formation of a boundary protective film primarily composed of oxides and MoS_2_ during friction, this film has low shear strength and high load-bearing capacity, thereby improving the friction-reducing and wear-resistant performance of the grease. Oily additive molecules generally have a long chain structure with a polar group at one end and a non-polar hydrocarbon-based chain at the other end [116]. Polar groups have a strong affinity for metal surfaces and can be tightly adsorbed on metal surfaces to form a molecular film with a directional arrangement [117]. This film has good flexibility and lubricity, which can effectively reduce the friction between metal surfaces even under low load and low speed working conditions. Due to its intermolecular forces, it can form a more stable lubrication layer on the surface of the parts, which has the effect of reducing friction and wear [118].

Hou et al. [119] studied the preparation of potassium borate/graphene (PB/GN) nanocomposite grease additives suitable for marine diesel engines. The study was carried out by pin-on-disc friction tester at various loads and temperatures, and the friction surface was comprehensively analyzed by scanning electron microscope and energy spectrometer. The results showed that the tribological properties of the base oil were enhanced due to the synergistic effect of PB and GN.

Anti-wear polymer additives are composed of high-molecular-weight polymers and exist in a dispersed state in gel-like lubricating grease. When mechanical parts move relative to each other, these polymer molecules form a physically adsorbed film on the metal surface, filling in microscopic irregularities on the surface and making it smoother. In addition, during friction, polymer molecules undergo deformation and orientation, further enhancing the anti-wear performance of the film. At the same time, it can also work synergistically with other additives to improve the overall anti-wear effect of the gel-like lubricant. Zhao et al. [120] found that molybdenum dialkyl dithiocarbamate (MoDTC) exhibits excellent anti-friction and anti-wear properties when used as an additive in aluminum-based gel-type lubricating grease, and exhibits significant synergistic effects with ZDDP. During friction, the two form a composite boundary lubrication film rich in phosphates/pyrophosphates and MoS_2_, with the P element promoting the conversion of MoDTC to MoS_2_, thereby enhancing lubrication performance. This composite film has low shear strength and high wear resistance, with synergistic effects superior to those of a single additive. The shape of nanoparticles can effectively enhance tribological performance, reducing friction and wear. Kamel et al. [121] prepared a calcium-based gel-like lubricant using graphene nanosheets (GNs) and TiO_2_ as additives. The study found that GNs and TiO_2_ exhibit synergistic effects in the lubricant, with friction-reducing and wear-resistant properties improved by 46.7% and 43.9%, respectively. Xia et al. [122] investigated the performance of MoS_2_/AlOOH nanocomposites as lubrication additives. The results showed that MoS_2_ was assembled in situ on the surface of AlOOH, which significantly improved the dispersion and optimized the lubrication performance. At the optimal addition concentration of 0.5 wt%, the average coefficient of friction was reduced by 50.47%, the diameter of abrasion marks was reduced by 42.34%, and the amount of wear was drastically reduced by 86.57% compared with that of the base oil. The lubrication mechanism lies in the formation of highly dispersive lubricant film by the nanocomposite structure during the friction process, which reduces friction and wear.

Research indicates that the molecular structure design of additives, the synergistic effects of multi-component systems, and the interfacial film-forming mechanisms are central to optimizing the performance of lubricating greases, providing a critical technological pathway for the development of high-performance lubricating materials under extreme operating conditions.

Nano-additives enhance the anti-wear performance of gel-like lubricants through multi-scale synergistic effects. At the microscopic level, hard nano-particles form a supportive structure, while layered nano-materials create a transfer film, reducing surface contact and shear stress. The three-dimensional network of the gel matrix provides a stable dispersion environment for the nano-particles, and its viscoelastic properties aid in transporting the particles to the friction interface. During friction, nanoparticles repair surface damage, and some active materials undergo friction chemical reactions with metal to form protective lubricating films. This multi-mechanism synergy enhances the grease’s extreme pressure performance and durability. The dispersion state, particle size, and surface chemical properties of nanoparticles are key factors determining anti-wear performance, providing guidance for optimizing grease formulations.

Table 4 summarizes the reduction in friction coefficients under the influence of different types of nano-additives when complying with ISO standards.

### 4.2. Friction Reduction

Gel-like lubricant nano-additives have unique advantages in reducing friction. They significantly improve grease performance through multiple mechanisms, reducing friction loss between mechanical components [128]. The microscopic filling and smoothing effects of nanoparticles can fill microscopic defects [129]. The surfaces of mechanical components are not completely smooth at the microscopic level, with numerous tiny bumps, depressions and scratches [130]. The nanoparticles in gel-like lubricant nanoadditives are extremely small, typically ranging from a few nanometers to several hundred nanometers, and can easily fill these microscopic defects [131]. Silica nanoparticles can be embedded in tiny grooves on metal surfaces, flattening an otherwise rough surface on a microscopic scale [132]. This microscopic flattening reduces the direct contact area and mutual occlusion between the surfaces when the parts are moving relative to each other, thus reducing the frictional resistance [133].

The addition of nano-additives to gel-like lubricants creates a microscopic rolling bearing effect, with some spherical nanoparticles, such as nano-metal balls and nano-ceramic balls, dispersing well in the lubricant [134]. These nanospheres can act like rolling bearings between the surfaces of the parts when the mechanical parts slide relative to each other [135]. They partially convert the original sliding friction into rolling friction, which has a much lower coefficient of friction than sliding friction [136]. As an example, between two metal surfaces in relative motion, copper nanospheres can roll freely, greatly reducing the frictional resistance between components and reducing energy loss chemical reaction film formation to reduce friction [137]. Reactions of active nanoadditives: Some gel-like lubricant nanoadditives are chemically active and contain nano-compounds of elements such as sulfur, phosphorus, and boron [138]. These active nano-additives react chemically with the metal surface during friction when the surface temperature of the mechanical component increases [41]. Nanosulfides react with iron atoms on metal surfaces under high temperature and friction to form iron sulfide films [139]. This iron sulfide film has low shear strength and acts like a smooth lubricant, effectively reducing the coefficient of friction between parts and allowing for smoother relative motion. Meng et al. [140] employed an aldehyde reduction method in supercritical carbon dioxide fluid to load silver nanoparticles (Ag NPs) onto the outer walls of multi-walled carbon nanotubes (MWCNTs), thereby preparing Ag/MWCNT nanocomposites. During friction, MWCNTs play a unique role, as their tubular structure facilitates surface sliding during friction; meanwhile, the nanoscale Ag particles utilize their high ductility to deform under friction pressure and fill the microscopic pores that MWCNTs cannot cover, forming a dense protective layer. Additionally, the low shear strength of Ag reduces friction resistance between the layers. The two components exhibit a synergistic effect, achieving lubrication through “structural complementary repair” rather than ‘rolling’ or “grinding.” MWCNTs provide skeletal support, while Ag NPs fill in detailed defects, jointly constructing a smooth and wear-resistant protective film. Through four-ball friction tests, it was found that adding 0.18 wt% of this nanocomposite reduced the friction coefficient of engine oil by 36.4% and decreased the diameter of wear marks by 32.4%. The supercritical CO_2_ synthesis method ensures uniform dispersion of silver nanoparticles, which synergistically form an efficient lubricating protective film with MWCNTs, significantly reducing friction and wear. Zhao et al. [141] found that MoS_2_ directly adhered to the steel surface to form a solid lubricating film, which synergized with ZDDP to generate a composite friction film and enhance surface protection. PP grease with additives maintains stable viscoelasticity, good viscosity recovery and excellent ductility at low temperatures. Min et al. [142] prepared a suspension of carbon nanotube microspheres consisting of carbon nanotube particles and sunflower oil by using the water-in-oil method, to which 0.01% by weight of carbon nanotubes and carbon nanotube microspheres were added, respectively. The results showed that the CNT microspheres exhibited low friction of about 0.11 over 5000 sliding cycles compared to the base oil. The formation and function of self-healing film, certain nano additives are able to repair tiny damages on metal surfaces during friction, forming a self-healing film. Under the action of frictional heat and pressure, copper nano-additives will be deposited on the metal surface and diffusion will occur, filling tiny wear pits and scratches to form a dense copper film. This self-healing film not only repairs the surface damage, but also has good lubrication properties, further reducing friction and improving the wear resistance and service life of the part. Zhai et al. [123] prepared three forms of graphitic carbon nitride (g-C_3_N_4_): block-shaped (CNB), plate-shaped (CNS), and tubular-shaped (CNNT). The results showed that adding 0.06 wt% CNNT lithium-based grease reduced the average scratch diameter and average friction coefficient by 28% and 10%, respectively. Due to the stable high dispersion of CNNT in the grease, an effective lubricating film is formed on the friction pair surface. By improving the rheological properties of the grease, nano-additives can significantly influence the rheological performance of the grease. They can increase the viscosity and viscosity stability of gel-like lubricant, enabling it to maintain good flowability and adhesion under various operating conditions. In low-temperature environments, nano-additives can prevent gel-like lubricants from becoming too viscous, ensuring that they can smoothly reach all lubrication points and reduce friction resistance during startup [143]. In high-temperature and high-load conditions, nano-additives can also maintain sufficient viscosity in gel-like lubricants, preventing lubrication failure due to the lubricant becoming too thin, thereby effectively reducing friction. Yang [39] chose 3# general-purpose lithium grease and 7014 wide-temperature aviation grease as the base grease, and selected nano-SiO_2_ and nano-TiO_2_ particles as the additives to analyze the reasons for the decrease of apparent viscosity with shear rate from the perspective of colloid chemistry, and to analyze the lubrication mechanism of nano-particles. And the test was carried out on the grease containing nanoparticles with a rheometer, and the rheological equation was fitted and the Reynolds equation was deduced, and the results showed that the apparent viscosity decreased with the increase of shear rate, and it had obvious shear-thinning property and significant non-Newtonian characteristics. Xu et al. [144] used the carbonization method to prepare three different particle sizes of nano-calcium carbonate (CaCO_3_) particles, and characterized their morphology and structure using transmission electron microscopy (TEM) and X-ray diffraction (XRD). All nano-CaCO_3_ particles exhibit a calcite crystal structure, but differ in particle size. The results indicate that nano-particles significantly enhance the anti-friction and wear-resistant properties of base lubricating grease. Additive concentration and particle size are key influencing factors, and optimal ratios must be optimized to achieve the best results. The addition of nano-particles facilitates the uniform dispersion of other additives in gel-based lubricating grease. Nanoparticles can serve as carriers to distribute traditional extreme pressure anti-wear agents, oiliness agents, and other additives more uniformly throughout the gel-like lubricant system. This allows various additives to synergize more effectively during friction, maximizing lubrication performance and further reducing the coefficient of friction. Li et al. [145] used polyamide (PA6) as an additive for lithium-based grease, employing thermogravimetric analysis and infrared spectroscopy to characterize and analyze the physical and chemical properties of polymer particles, and conducted characterization analysis of the wear scar region. The results showed that the PA6+LBG grease had the lowest average friction coefficient at a load of 20 N and a speed of 50 mm/s, with a 57.4% reduction in friction coefficient and a 74.7% reduction in wear volume compared to lithium-based grease. This section focuses on the application of nano-additives in reducing friction in gel-like lubricants, explaining how they enhance lubrication performance through multiple mechanisms, including composite synergy, self-repair and morphology control, rheology and interface optimization, providing theoretical support for the development of high-performance lubricants.

The mechanism by which nano-additives enhance anti-friction performance in gel-like lubricants is a complex process involving multi-scale synergistic interactions. At the microscopic level, nano-additives, with their unique geometric shapes and size effects, can form a uniform nano-scale lubricating film at the friction interface. This ultra-thin lubricating film ensures good adhesion to the contact surface while significantly reducing the coefficient of friction due to its low shear strength. More importantly, spherical or near-spherical nanoparticles generate a “micro-bearing” effect in the contact area, converting part of the sliding friction into rolling friction. This mechanism effectively reduces friction resistance and wear rate. Additionally, certain nanomaterials with special structures exhibit excellent self-healing properties, dynamically repairing microscopic defects formed on worn surfaces through surface diffusion or chemical reactions, thereby maintaining the integrity of the friction interface. The oriented arrangement behavior of layered nanomaterials under shear force forms a highly ordered lubrication layer, and this ordered structure further optimizes the energy dissipation characteristics of the friction interface. Furthermore, the surface chemical properties of nanoparticles influence their interaction with metal surfaces, thereby significantly impacting the overall anti-friction performance. This multi-mechanism synergistic anti-friction system enables nano-composite lubricants to maintain excellent lubrication performance while significantly extending equipment lifespan, providing important insights for developing the next generation of high-performance lubrication materials. Table 5 summarizes the wear scar diameters corresponding to different types of nano-additives under ISO standard testing.

### 4.3. Oxidation Resistance

Oxidation is a common chemical reaction, but it can cause harm to substances and the environment, etc., and reduce the mechanical properties [146], and the oxidized layer formed on the surface of the metal after oxidation reaction is usually loose and fragile in texture, which can destroy the original compact structure of the metal [147]. The generation of rust after rusting of iron (the main component is iron trioxide, etc.) will make the strength, toughness and other mechanical properties of iron decrease dramatically, leading to easy fracture and deformation of metal products, affecting the safety of its use and life [148]. Antioxidants are mainly added to the base grease to prolong the service life of the lubricant by retarding the aging process caused by oxidation of the base grease molecules [149]. Xue et al. [150] investigated the antioxidant effect of an arylamine oligomer antioxidant in base oil (ester), lithium grease by pressurized differential scanning calorimetry and oven oxidation, and the performance of the antioxidant in AS5780 aviation turbine engine oil by oxidation stability and corrosion tests. The results showed that the aromatic amine oligomer antioxidant has outstanding antioxidant effect in base oil (ester). Excellent high-temperature antioxidant performance was demonstrated in both grease and aviation turbine engine oil. The incorporation of this oxidizing agent can prolong the service life of the machine.

It has been shown that hydrotalcite materials have good photo-thermal stability and are green and non-toxic, and are used as anti-aging agents in polymer materials and have a tendency to dehydrogenate, so they have the potential to be used as antioxidants in lubricating greases [151]. Jin [30] investigated hydrotalcite material as an antioxidant for lubricating grease by inserting traditional thickeners dodecahydroxystearic acid (12HSA) and stearic acid (SA) into the hydrotalcite interlayer to obtain Mg_2_Al(12HSA)-LDH and Mg_2_Al(SA)-LDH, which were mixed with two different types of base oils, and the lubricating oils were thickened into grease by powerful ultrasonication. The results showed that the thickening performance was enhanced with the increase of the addition amount. Hydrotalcite has the potential to be used as a new type of grease thickener. “Dual-functional ligand design” has brought innovative breakthroughs to nanoparticle surface engineering. Taking superparamagnetic iron oxide (USPIONs) as an example, the dual-functional system constructed using a 2PG-S*VVVT-PEG4-ol peptide coating demonstrates excellent performance. The phosphate groups in the coating can be firmly anchored to the surface of iron oxide particles through chemical bonding interactions, forming stable binding sites; simultaneously, the PEG segments create a physical barrier around the particles via steric hindrance effects. This dual-functional coating enhances the stability of USPIONs in electrolyte solutions by threefold, effectively preventing aggregation caused by protein adsorption and ion complexation in physiological environments. It significantly improves the dispersion and stability of nanoparticles in complex systems, laying a solid foundation for their application in biomedical and environmental remediation fields. It can significantly enhance the dispersion and stability of nanoparticles in complex systems [38].

Zhang et al. [43] investigated the high temperature (210 °C) antioxidant properties of Fucene in polyurea grease (semi-synthetic oil with paraffinic base oil + PAO as base oil) using high pressure differential scanning calorimetry (PDSC). The oxidation induction period of the polyurea grease was 0 min (already oxidized below 210 °C) at 210 °C, while the oxidation induction period of the polyurea grease with 2.0% Fucene added was 11.5 min. The results showed that Fucene can significantly improve the high-temperature oxidation stability of the polyurea grease, and it has excellent high-temperature antioxidant performance. Chen et al. [152] investigated the effects of co-dispersant, temperature and antioxidant on the consistency of bentonite grease, the results showed that a heating temperature of 40 °C is a suitable synthesis temperature, and the characterization of the properties and structure of bentonite grease showed that bentonite grease with good thermal stability and antioxidant properties can be prepared under the condition of using acetone as a polar co-dispersant and amine additives as antioxidants. Wang et al. [153] synthesized four calcium composite sulfonate-based greases with different components for rust prevention and lubrication of mechanical equipment, and the four compounds were characterized using infrared chromatography. Salt spray, four-ball friction wear and rotary oxygen bomb tests were used to study the effects of rust prevention, oxygen resistance, extreme pressure anti-wear and rust prevention lubricants formulated with the addition of calcium sulfonate complex greases on their rust prevention, oxidation resistance and friction wear properties. The results show that the calcium sulfonate compound grease can meet the requirements for rust prevention and lubrication under various working conditions. This section focuses on research into antioxidant and multifunctional additives for lubricating greases. It was found that aromatic amine oligomers and Fukkenene antioxidants can improve high-temperature oxidation stability, while hydrotalcite has both anti-aging and thickening potential. Composite calcium sulfonate-based lubricating greases offer comprehensive performance in terms of rust prevention, oxidation resistance, and extreme pressure anti-wear properties, providing new insights for the formulation design of high-performance lubricating greases under high-temperature and multi-condition operating environments.

The enhanced antioxidant performance of nano-additives in gel-like lubricants primarily stems from their unique physicochemical properties and synergistic interaction mechanisms with the gel system. Nano-materials enhance the oxidative stability of grease through multiple pathways. First, their high specific surface area and surface active sites enable them to effectively capture and neutralize free radicals generated during oxidation. Second, their unique microstructure forms a physical barrier layer that inhibits oxygen diffusion and the propagation of oxidative reactions. Additionally, their excellent thermal conductivity aids in promptly dissipating friction heat, thereby reducing the risk of thermal oxidation. The interface interactions between nanoparticles and the gel matrix further optimize their dispersion state and activity maintenance, while surface functionalization modifications confer the ability to intelligently release antioxidant components. This multi-scale, multi-dimensional synergistic protective mechanism enables composite lubricants to maintain stable antioxidant performance under harsh conditions such as high temperature and pressure, significantly extending the service life of lubrication systems, and providing new insights for the design of high-performance nano-additives in gel-like lubricants.

### 4.4. Load Carrying Capacity

**Figure 13:**
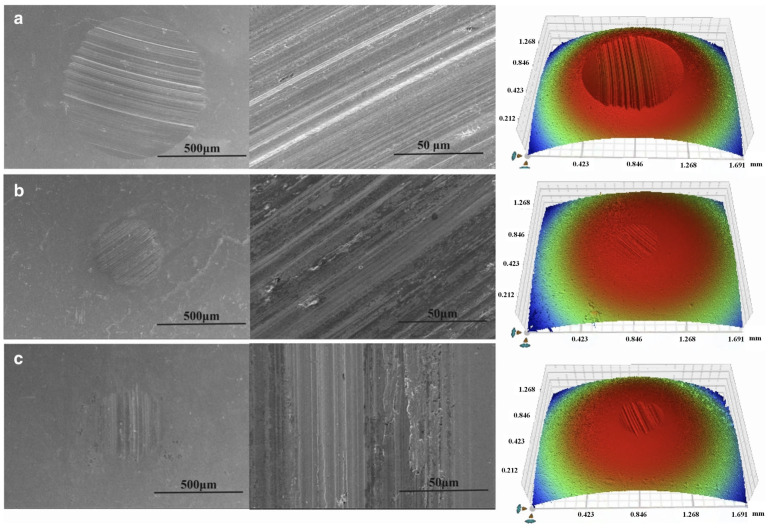
Semiconductor Electron Microscope (SEM) image and three-dimensional (3D) morphology of the worn steel surface under different lubricant conditions: (**a**) PAO lubricant, (**b**) PAO/0.2 wt% OM-modified CeO_2_ nanoparticle lubricant, (**c**) PAO/1.8 wt% OM-modified CeO_2_ nanoparticle lubricant (load: 392 N; speed: 1200 rev/min; time: 60 min; temperature: 75 °C) (Reprinted with permission from Reference [156]. Copyright 2020 Springer).

Nano-additives significantly enhance the load-carrying capacity of gel-like lubricants through multiple mechanisms [154], forming a protective film. The potential friction-chemical interactions primarily involve nano-scale rolling, repairing, or polishing mechanisms. Nano-rolling mechanism: Under high pressure and shear stress during friction contact, materials (which may be decomposition products of additives, base oil molecules, or even the base metal itself) undergo plastic deformation or rolling, forming tiny spherical or ellipsoidal particles. These particles roll between the friction surfaces like “micro-bearings”, converting sliding friction into rolling friction, thereby significantly reducing the coefficient of friction and wear. Nano-repair mechanism: Active substances (wear particles, additive reaction products) or the material itself generated during friction are “pressed” or “welded” onto the worn surface (especially defects such as micro-cracks and micro-pits) under the high temperature and pressure of friction contact, filling the damaged areas, restoring the surface to a smooth state, preventing crack propagation, and thereby reducing the wear rate. Nano-polishing mechanism: Extremely fine hard particles (such as oxides, carbides) or low shear strength layers formed by additives during friction act like tiny “grinding tools” during relative sliding, continuously and gently removing microscopic protrusions (micro-asperities) from the friction surface. This gentle grinding action makes the contact surface smoother, increases the actual contact area, reduces contact stress, and thereby reduces friction and wear. Nanoparticles (such as nano-silica, nano-zirconia, etc.) can form a uniform nano-protective film on the friction surface, reducing direct contact between friction pairs, lowering the friction coefficient, and reducing wear rates [155]. Wu et al. [156] prepared cerium oxide nanoparticles modified with oleylamine using one-pot pyrolysis method. The tribological properties were investigated using a four-ball machine, and the results showed that the nanoparticles displayed good dispersion in PAO as well as excellent anti-wear ability. The modified CeO_2_ nanoparticles can catalyze the oxidation of metallic iron to form a friction film containing ferrite, which has a greater load-bearing capacity. As shown in Figure 13.

To improve structural strength, nanoparticles can enhance the structural strength of gel-like lubricants, enabling them to maintain good lubrication performance even under high load conditions. Nano-silica can significantly increase the yield stress and flow stress of lubricants [157]. Zhou [158] analyzed the tribological properties of nanocomposite calcium-titanium sulfonate-based grease under various working conditions through extreme pressure antiwear tests and high temperature bench experiments, and found that nano-WS_2_/MoS_2_ composite particles have less effect on the lubrication performance of grease under low temperature and low load, while it can significantly improve the tribological performance of grease and prolong its service life under high temperature and heavy load. Different types of nanoparticles (e.g., metal oxides, carbon nanomaterials, etc.) can act synergistically to further enhance the comprehensive performance of the grease [159]. The combined use of nano-titanium dioxide and nano-silica can significantly improve the load-bearing capacity and anti-wear performance of gel-like lubricant [160]. Sun et al. [161] used a rheometer and Optimol SRV-V friction tester to investigate the rheological and tribological properties of calcium sulfonate greases containing micron/nano calcium carbonate (CaCO_3_) additives at 30 °C and 80 °C. The results showed that the grease with 5% micronized CaCO_3_ additive exhibited superior friction reduction and antiwear properties than the grease with 5% nanometer CaCO_3_ additive under boundary lubrication conditions. The micronized CaCO_3_ synergistically interacted with the intrinsic CaCO_3_ in the calcium sulfonate grease to generate a CaCO_3_/CaO composite film, which effectively reduced friction and wear. Through techniques such as surface modification, the nanoparticles can be uniformly dispersed in the grease to avoid agglomeration, thus giving full play to their performance [162]. Cao et al. [124] dispersed graphene nanoparticles (GNPs) in oleic acid for mechanical milling, and utilized oleic acid for surface modification modification of the GNPs, which were added to a composite lithium grease. The results showed that the addition of a small amount of GNPs could significantly enhance the high temperature and tribological properties of the grease. The best lubrication effect was achieved when the mass fraction of GNPs was 0.15%, and compared with the base grease, the diameter of the abrasive spot and the minimum average coefficient of friction were reduced by 20.5% and 32.8%, respectively, and the extreme pressure performance was improved by 43.7%. This section focuses on the application of nano-additives in gel-like lubricants. Research indicates that material modification, size control, and surface modification techniques for nano-additives are key to overcoming performance bottlenecks in lubrication under complex conditions such as high and low temperatures and heavy loads, thereby providing technical support for the development of high-performance lubricant materials.

The enhanced load-carrying capacity of nano-additives in gel-like lubricants primarily relies on their unique microscopic mechanisms of action. Nano-particles form strong interfacial bonds with the gel matrix through surface modification, and their high specific surface area effectively disperses contact stress. Meanwhile, the sliding mechanism of layered nano-materials helps buffer local pressure. The appropriate addition of nanomaterials can significantly improve the extreme pressure performance of gel-like lubricants, which may be attributed to the supportive structures and self-healing protective films formed by nanoparticles in the friction contact zone. It is important to note that the mechanism of action of nanoadditives under extreme friction conditions is highly complex, involving processes such as particle transport, adsorption, potential deformation, or chemical reactions in the contact zone. The resulting interface structure may not be fully captured by the simple concept of a homogeneous “film”. This study has not yet systematically established a clear quantitative relationship between the key parameters of nanoparticles (such as size and concentration) and their dispersion stability in grease, the characteristics of the protective structures formed at the friction interface, and their tribological performance. This is primarily due to the complex, potentially nonlinear interactions among various factors (particle size, concentration, surface chemistry, dispersion state, base grease properties, and friction conditions), making it extremely challenging to isolate the influence of a single variable. A deeper understanding of the interdependent relationships among these factors is crucial for precisely controlling the performance of nano-additives, optimizing formulation design, and elucidating their fundamental lubrication mechanisms. This is also the direction we should pursue in future research. Table 6 summarizes the load-bearing capacities of different types of nanoadditives based on ISO standard testing.

### 4.5. Surface Analysis Technology

**Figure 14:**
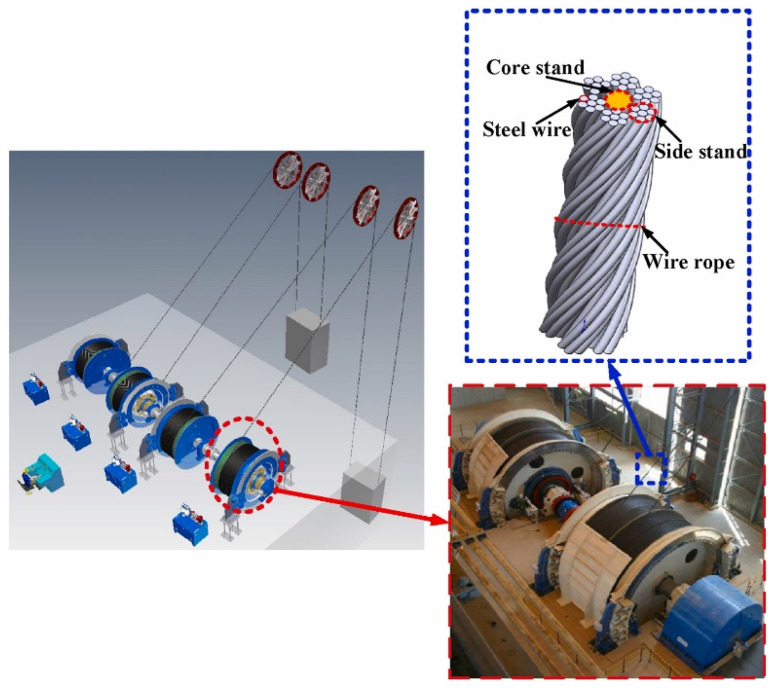
Multi-layer winding crane. (Reprinted with permission from Reference [164]. Copyright 2022 Elsevier).

**Figure 15:**
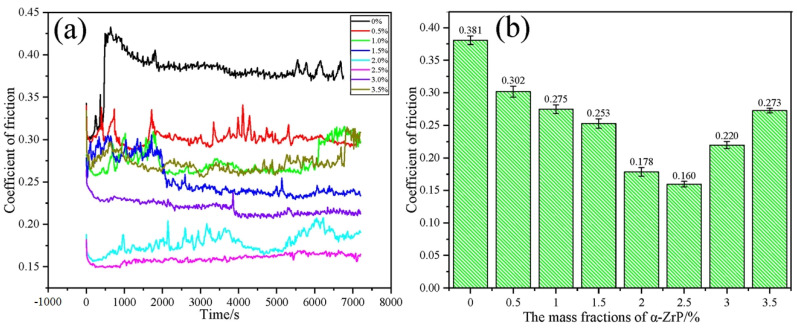
The impact of α-ZrP admixtures with various mass fractions on the change in the coefficient of friction (CoF) of steel wire ropes over time. (**a**) Coefficient of friction (CoF); (**b**) Average coefficient of friction (CoF). (Reprinted with permission from Reference [164]. Copyright 2022 Elsevier).

Surface analytical techniques are the core means to study the composition, structure, chemical state and physical properties of material surfaces (from atomic to micrometer scale). It has an irreplaceable role in scientific research, industrial production and quality control [163]. This section focuses on a systematic evaluation study of the performance of gel-like lubricant nanoadditives. To comprehensively analyze key performance indicators such as microstructure and elemental composition, a variety of advanced characterization techniques were employed during the experiments, including scanning electron microscopy (SEM), transmission electron microscopy (TEM), X-ray photoelectron spectroscopy (XPS), atomic force microscopy (AFM), and Raman spectroscopy. Among these, scanning electron microscopy (SEM) enables high-resolution observation of the surface morphology of the sample; transmission electron microscopy (TEM) provides detailed analysis of the internal microstructure and crystal structure information of the sample; X-ray photoelectron spectroscopy (XPS) is used to precisely determine the elemental composition, chemical valence, and content of the sample surface; Atomic force microscopy (AFM) enables three-dimensional topographical imaging and mechanical property analysis of sample surfaces at the nanoscale; Raman spectroscopy detects molecular vibration spectra to effectively characterize the chemical structure, phase state, and intermolecular interactions of samples. Rawat et al. [64] investigated the effect of CuO and ZnO nano-additives on the tribological properties of paraffinic oil based grease, dispersion of ZnO and CuO nano-additives in paraffinic grease reduced the energy consumption by about 31% and about 28%. Wang et al. [82] utilized the four-ball test to assess the tribological properties of additives in lubricating grease at different concentrations and ratios. An artificial neural network (ANN) model was established. Genetic algorithms were used to predict the optimal concentrations of multiple additives in lubricating grease, which were then experimentally validated. The results showed that introducing FGR (0.14 wt%) and FCNT (0. At an addition rate of 16 wt%, the anti-friction and anti-wear properties of the base lubricating grease improved by 25.66% and 29.34%. Zhang et al. [164] evaluated the tribological properties of α-ZrP (layered zirconium phosphate) modified grease by using four-ball friction tester, steel wire micromotor wear test rig and wire rope sliding wear test rig. As shown in Figure 14. it was found that the coefficient of friction (CoF) increased and then decreased with α-ZrP addition. When the α-ZrP addition is 2.5 wt%, the grease has the best protective effect on the mine hoisting wire rope, significantly reducing friction and wear. Its laminar structure can form an easy shear film in the friction process and improve the lubrication performance, but the excessive addition will lead to particle agglomeration, but not conducive to lubrication, as shown in Figure 15.

**Figure 16:**
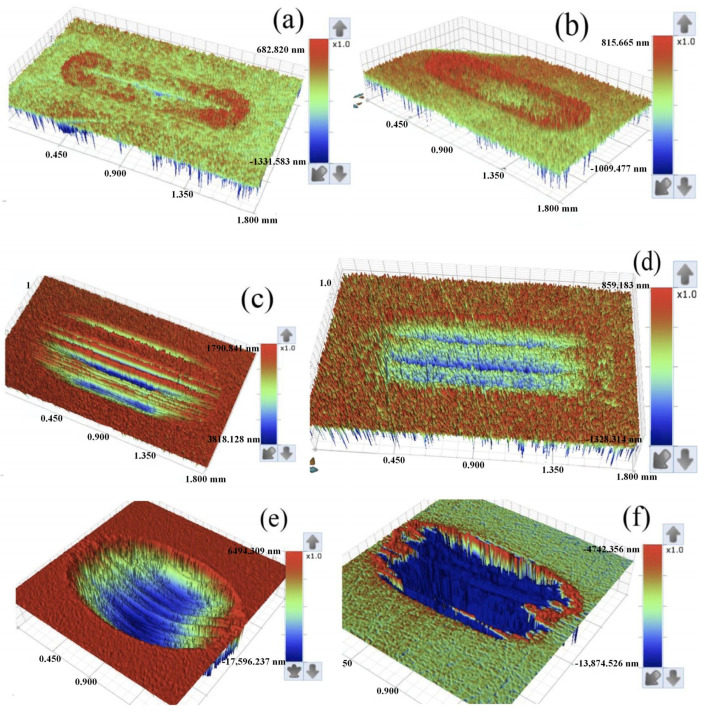
Surface morphology of the wear surface under different loads: (**a**) Base Grease and (**b**) Base Grease + 0.5% GN at 100 N load; (**c**) Base Grease and (**d**) Base Grease + 0.5% GN at 200 N load; (**e**) Basis Grease and (**f**) Basis Grease + 0.5% GN at 400 N load. (Reprinted with permission from Reference [104]. Copyright 2018 Elsevier).

Du et al. [63] found that when the TiO_2_ to CeO_2_ ratio was 6:4, SEM observations showed an average particle size of 45 nm, a standard deviation of 8 nm, and uniform dispersion. However, at ratios of 7:3 and 5:5, the particle sizes reached 60 nm and 55 nm, respectively, with agglomerates ranging from 200 to 300 nm in size; XRD analysis indicated that this ratio formed a stable composite phase with a lattice distortion rate of 0.3%, while the 7:3 ratio had a distortion rate of 0.8% and contained amorphous impurities; In terms of friction performance, the 6:4 ratio exhibits a friction coefficient of 0.08 (a 28.6% reduction compared to 7:3) and a scratch diameter of 0.35 mm (a 26.3% reduction compared to 5:5). The surface forms an oxide film with a thickness of 80–100 nm and contains new phases such as FeTiO_3_. Other ratios exhibit poorer performance due to incomplete film formation. Niu et al. [165] studied the tribological properties of zirconium phosphate-quinoline compounds (ZrPOF-Q1) as additives for lithium-based greases. The core mechanism by which ZrPOF-Q1 enhances lubrication performance lies in its unique film-forming mechanism: the phosphate groups (PO_4_^3−^) in ZrPOF-Q1 may react with Fe^2+^/Fe^3+^ on the metal surface to form iron phosphate salts, while the nitrogen atoms in the quinoline rings form coordination bonds with the metal through lone pair electrons. Together, these components form a chemically adsorbed film that fills nanoscale defects on the surface. The film formation of ZrPOF-Q1 relies on chemical bonding (such as metal-oxygen bonds and coordination bonds), which ensures a stronger bond between the film layer and the substrate. This allows it to remain stable under extreme pressure conditions, which is precisely why it exhibits “enhanced extreme pressure performance”. Through four-ball friction testing machines and surface analysis techniques (3D optical profilometer, SEM, EDS), it was found that ZrPOF-Q1 significantly enhances the anti-wear, friction-reducing, and extreme pressure performance of lubricants. During friction, a protective film containing ZrPOF-Q1 reaction products forms, effectively improving lubrication performance. Bas et al. [105] used XRD, SEM, profilometer, and EDS to investigate the effects of MoS_2_ and CaF_2_ additives on the tribological properties of lithium-based grease by forming composite protective films: XRD detected the formation of stable crystalline phases and reaction products on the worn surface; SEM observations showed a reduction in surface grooves, elimination of adhesive wear, and the formation of a continuous film with a thickness of 50–80 nm; profilometer measurements indicated a 75% reduction in surface roughness, with the friction coefficient decreasing from 0.14 to 0.08 (a 43% reduction); EDS confirmed uniform element distribution. Ultimately, wear rate was reduced by 58%, and the PB value increased by 42%. Zhao et al. [42] used the Optimol-SRVIV oscillating friction and wear tester (SRV tester) to evaluate the tribological properties of nanocarbon-based materials (NCB) as additives in lithium-based greases. During the friction process, NCB exhibits a unique lubrication mechanism: NCB nanosheets or tubular structures align and deposit along the metal surface under the action of friction shear force, forming a protective film similar to a “coating”. This alignment reduces surface energy while leveraging the inherent lubricity of carbon materials (such as the low friction characteristics of sp^2^ hybridized carbon) to achieve friction reduction. This is the physical film-forming process of the carbon-based film. Unlike the polishing mechanism, which achieves surface smoothness at the cost of “material removal”, NCB’s repair mechanism centers on “material deposition”, optimizing the surface by filling defects. When layered carbon-based materials such as graphene and molybdenum disulfide are used as additives, they typically function through “physical adsorption film formation + defect filling”, falling under the category of nano-repair mechanisms, which aligns with NCB’s film-forming logic. The results show that NCB significantly enhances the anti-wear performance, load-bearing capacity, and friction-reducing effects of lubricating grease. SEM observations reveal improved surface morphology of the worn surface, and XPS analysis confirms the formation of a carbon-based protective film by NCB during the friction process. Kumar et al. [103] analyzed OA-LaF_3_ and COOH-MWCNTs using various techniques: HR-TEM revealed that the former was nearly spherical (average particle size 15 nm) and the latter was hollow tubular; XRD determined that OA-LaF_3_ was a hexagonal crystal (lattice distortion rate 0.3%); Fourier Transform Infrared Spectroscopy (FTIR) detected characteristic functional groups in both; XPS analyzed elemental composition and wear surface friction films; SEM observed the smoothest wear surface in the 0.075 wt% OA-LaF_3_ group; EDS identified corresponding elements. The results confirm that both additives improve lubrication performance by forming a friction film. The 0.075 wt% OA-LaF_3_ formulation reduced the coefficient of friction by 43.5% and surface roughness by 83%, while the 0.05 wt% COOH-MWCNTs formulation reduced the diameter of wear marks by 19%. Wang et al. [104] used X-ray photoelectron spectroscopy (XPS) and Raman spectroscopy to examine the wear surface to study the tribological mechanism. The results showed that the wear resistance and friction reduction of the grease was significantly improved due to the incorporation of graphene, as shown in Figure 16.

Mohamed et al. [166] characterized the microstructure of the synthesized nanoparticles by X-ray diffraction (XRD) and transmission electron microscopy (TEM), and evaluated the tribological properties of the nanogrease using a four-ball friction tester.SEM observations showed a significant improvement in the morphology of the wear surfaces, and the EDX assay confirmed the presence of nanoparticles in the friction film with characteristic elements, which suggests that they are involved in the friction chemical reaction. Wu et al. [36] investigated the effect of alkyl chain length on the performance of CQD as a hybrid grease additive by using a four-ball friction and wear tester, scanning electron microscopy (SEM), energy dispersive X-ray spectroscopy (EDS) and X-ray photoelectron spectroscopy (XPS). It was shown that the coefficient of friction (COF), wear spot diameter and depth of abrasion were reduced by 33.65%, 42.11% and 51.87%, respectively, when the modified CQD with the shortest alkyl chain length was used compared with pure LG.

Surface technology research on nano-additives in gel-like lubricants primarily focuses on three key dimensions: interfacial interaction mechanisms, surface characterization techniques, and performance optimization methods. In terms of interfacial interactions, stable bonding between nanoparticles and the gel matrix is achieved through multiple mechanisms such as physical adsorption, chemical bonding, and electrostatic interactions. Surface modification and in-situ modification techniques are employed to regulate interfacial compatibility. Surface morphology and chemical analysis are systematically studied using advanced characterization techniques such as SEM, AFM, and XPS to investigate the dispersion state of nano-additives, interfacial chemical composition, and stress distribution characteristics. Performance optimization is achieved through improved dispersion processes, surface texturing design, and environmentally responsive modifications, significantly enhancing the tribological performance and service stability of gel-like lubricants. Proper surface treatment enables nano-additives to form a stable dispersion system within the gel matrix, constructing an effective nano-lubricating film at the friction interface, thereby improving the anti-wear and friction-reducing performance, environmental adaptability, and service life of gel-like lubricants. These surface technology research findings provide important theoretical guidance and technical support for the development of high-performance nano-composite gel-like lubricants, demonstrating significant advantages in applications such as industrial bearings and precision machinery, particularly in surface protection and friction control under extreme operating conditions.

## 5. Application Scenarios for Nano-Additives in Gel-like Lubricants

### 5.1. Industrial Manufacturing

Along with the development of modern industrial technology, in industrial manufacturing, equipment is often faced with demanding working conditions of high loads, high temperatures, and high speeds [167]. Such as gearboxes in heavy-duty machinery, precision reducers, and even the main shaft bearings of wind turbines. These scenarios place extremely high demands on gel-like lubricants, while traditional lubricants often fail under high temperatures and pressures, leading to increased equipment wear and reduced service life [168]. The main shaft bearings of offshore wind turbines are subjected to long-term alternating loads (wind speed fluctuations) and salt spray corrosion, and the maintenance cost is extremely high (more than 500,000 RMB for a single lifting) [169]. The use of traditional grease is prone to salt spray infiltration that triggers bearing corrosion, and leads to grease delamination at extreme temperature differences (−30 °C to 80 °C). It will increase the wear and tear of the spindle bearings and reduce the life of their use [170]. With zinc oxide (ZnO) nano-additives, ZnO nanorods adsorb chloride ions, inhibit galvanic corrosion, and increase the duration of their use [171]. The maintenance interval of main shaft bearings in an offshore wind farm was extended from 6 months to 2 years after the addition of nano-additives. And the failure rate of the bearings decreased by 70%, and the annual loss of power generation was directly reduced by 15% [172]. However, after adding nano-additives to gel-like lubricant, nano-zinc oxide may react with sulfides in the environment, so it is necessary to monitor the condition of the grease regularly [173]. Gel-like lubricant are also mainly used in industrial robot joint gearboxes. Six-axis robot joints require high-frequency reciprocating motions (tens of thousands of cycles per day), and precision gearboxes require extremely high grease cleanliness. Problems arising from the application of traditional grease are long-term shear leading to the destruction of the grease structure, resulting in metal debris contamination, triggering a decline in positioning accuracy. Nanodiamond particles and polytetrafluoroethylene (PTFE) microspheres are added to grease [174]. The mechanism of action is that nanodiamonds improve shear resistance and reduce grease rheological failure, while PTFE microspheres reduce the coefficient of friction and realize “silent lubrication” [175]. After adding nano-additives to gel-like lubricant, the maintenance-free cycle of a robot reducer on an electronic assembly line was extended from one year to three years. Furthermore, repeat positioning accuracy was maintained within ±0.02 mm (traditional grease: ±0.05 mm). However, the high hardness of nano-diamond may accelerate the wear of seals, which needs to be matched with ceramic-coated sealing rings [176]. High-speed CNC machine tool spindles have machine tool spindle speeds of 20,000~40,000 RPM, in which the grease added needs to have zero splash and low heat generation. However, the high-speed centrifugal force of traditional grease will make the grease fling out, and the temperature rise of the spindle is too high (over 90 °C), which triggers thermal deformation and is unfavorable to production. Adding nano-silica (SiO_2_) and ion liquid-modified nanoparticles to gel-like lubricant [177]. Silica forms a three-dimensional grid structure, enhancing the grease’s resistance to shedding. The result is a 35% reduction in temperature rise for precision spindles and improved machining accuracy to the micron level. The grease replenishment cycle has been extended from once a week to once a month. This significantly improves part performance. The emergence of gel-based grease nano-additives addresses issues that traditional greases cannot resolve [50]. For example, nanographene forms a protective film through a nano-repair mechanism. Its layered structure physically adsorbs and accumulates on friction surfaces, filling microscopic defects like “patches” and constructing a dense film layer with low shear strength, thereby achieving both friction reduction and wear resistance [178]. This protective film not only isolates the metal from direct contact, but also prolongs the life of the equipment by continuing to work at high temperatures and pressures. The screw of the injection molding machine generates local high temperature (200–250 °C) and high pressure (1000–2000 bar) during high speed rotation (100–300 RPM). The use of traditional grease in the screw will lead to carbonization of the grease under high temperature, resulting in guide jamming and increased wear of the screw. Increasing the danger of the machine in use. Assuming that the addition of additives boron nitride nanoparticles (h-BN) and titanium dioxide (TiO_2_) to the grease will delay the aging of the grease and enhance its antioxidant properties [179]. Its practical application in the automotive parts plant injection molding machine screw life will be increased from 6 months to 18 months. And the frequency of stopping for cleaning will be reduced by 60% and the productivity will be increased by 20%. However, in its practical application, nano-boron nitride is costly, and the price of a single kilogram is 8 to 10 times that of traditional grease, which increases the cost investment.

Nano-additives can also fill the tiny pits on the friction surface and act as a “repair” [40]. Du et al. [180] developed a novel bifunctional mesoporous silica nanocontainer for enhancing the self-repairing ability and friction reduction properties of polymeric materials. The nanocontainer was based on mesoporous silica nanoflowers loaded with copper nanoparticles (Cu NPs) as a solid lubricant and flaxseed oil as an external repairing agent.The Cu NPs provided friction reduction and the flaxseed oil realized wear repairing, and the synergistic combination of these two resulted in a self-repairing rate of up to 77%, which significantly prolonged the service life of the epoxy resin (EP) material. This repair is not a simple surface treatment, but allows the equipment to automatically “heal” during operation, reducing subsequent maintenance costs. Inspired by mussels, Chen et al. [50] realized in situ assembled PDA friction films using polydopamine (PDA) nanoparticles as aqueous lubricant additives. The results showed that the PDA friction film could form a chemical bond with the metal interface, and also produce a synergistic lubrication effect with the upper surface of ceramics. And the PAD friction film has self-healing effect. So it can realize ultra-stable lubrication performance during friction and effectively control friction and wear. Wang et al. [25] did tribological experiments by silver/graphene nanocomposites and concluded that their tribological performance is excellent, with the coefficient of friction and wear point diameter reduced by 40% and 36%, respectively. Moreover, the laminar structure of this composite has self-lubricating properties and the silver nanospheres can self-repair the lubrication film, which can significantly reduce friction and wear. Compared with commercial lubrication additives, it is more environmentally friendly and corrosion-resistant, and has a broad application prospect. Prado et al. [181] investigated the tribological properties of biodegradable polyester lubricants incorporating two different sizes of pristine graphene nanoplates. In this study, graphene nanoplates formed a protective film in the lubricant through a nano-repair mechanism. Their two-dimensional layered structure effectively filled microscopic defects on the friction surface via adsorption and stacking, thereby constructing a dense film layer with low shear strength, ultimately achieving friction reduction and wear resistance. The study concluded that the nano-lubricant formulated with GnP40 exhibited slightly better stability. Raman microscopy and wear surface roughness assessments revealed that the nano-lubricant demonstrated excellent tribological performance, attributed to the formation of this protective film and surface repair mechanism.

Additionally, the study indicated that lubricants containing nano-sized Cu, Al, Al_2_O_3_, and MgO particles all exhibit good friction reduction and repair capabilities on friction surfaces, but there are differences in the effects of each particle. The effects of Al_2_O_3_, Cu, and Al particles are superior to those of MgO, and mixed particles perform better than single particles. Nano-particles improve the tribological properties of gel-like lubricants through mechanisms such as micro-rolling, adsorption, filling, welding, and soft shear layers. However, graphene nano-sheets possess a unique nano-repair mechanism, enabling them to function in lubricants in ways that are both similar to and distinct from these nano-particles, collectively providing diverse pathways to enhance lubricant performance.

Ma et al. [182] studied the effects of different types of extreme pressure antiwear agents, antioxidants and rust inhibitors on the performance of lithium composite grease and prepared a lithium composite grease by compounding a multifunctional composite additive. The developed composite lithium grease has a Timken value of 266 N, an oxidation induction period of 76.1 min at high temperature, and a dropping point of more than 330 °C. The results show that the developed composite lithium grease has a high oxidation induction period of 76.1 min at high temperature. The results show that the developed lithium composite grease has excellent performance of extreme pressure anti-wear, high temperature anti-oxidation and bearing corrosion and wear resistance, and the application effect is good. This section focuses on the application of nano-additives in harsh industrial conditions, demonstrating how they significantly improve the performance of gel-like lubricants through mechanisms such as nano-repair and self-healing, thereby addressing the failure issues of traditional lubricants under high-temperature, heavy-load, and high-speed conditions. This provides a systematic solution for extending the maintenance cycle of industrial equipment and improving energy efficiency. Table 7 summarizes the advantages and disadvantages of traditional grease and gel-like lubricant in different applications.

### 5.2. Transportation

The demand for grease in the transportation sector is somewhat similar to that of industrial manufacturing, which focuses on basic requirements such as lubrication, friction reduction and anti-wear. For example, mechanical equipment in industrial manufacturing and vehicle equipment in transportation, they both need grease to reduce frictional resistance between friction parts, reduce energy consumption, and improve the efficiency of equipment operation and service life [184]. However, lubrication in the transportation sector has its own unique characteristics. Transportation equipment often operates in a variety of complex and changing outdoor environments, such as high temperatures, low temperatures, humidity, and dryness. As a result, the requirements for gel-like lubricants are even more stringent. Additionally, transportation equipment often operates continuously for extended periods of time, such as cars and trains that travel for long distances. This necessitates lubricants with longer service lives and more reliable lubrication performance to reduce maintenance frequency and downtime, thereby enhancing economic efficiency. For example, high-speed rail gearboxes not only need to operate stably at high speeds and high temperatures, but also must reduce noise and temperature rise, and reduce the frequency of maintenance. In addition, there is an urgent need to solve the problem of environmental protection in the field of transportation [185]. Guo et al. [186] constructed and validated a double-layer oil film lubrication model that integrates transient, thermal, and elastic effects, and systematically investigated the lubrication performance of roller floating bushings in diesel engines under different operating conditions and structural parameters. The study found that the lubrication conditions of the oil film inside this component are more severe, and the pressure it bears is significantly higher than that of the outer oil film. This result provides a key theoretical basis for optimizing the design of roller floating bushings in diesel engines and improving their reliability.

As transportation equipment operates outdoors, grease leakage or wear products are more likely to enter the natural environment directly and pollute soil and water bodies. Therefore, there is a need to develop environmentally friendly greases with higher biodegradability and lower environmental toxicity to minimize the negative impact on the environment. Realize the coordinated development of economy and environment. Conventional grease is poorly effective in the process of use and it is difficult to achieve the desired results. However, adding nano-additives to gel-like lubricants has shown good results. For example, the automobile engine parts of the working temperature difference is large and affected by the load, in order to meet the requirements of its special working conditions, need to use additives to improve the performance of the lubricant, with copper/graphene nano lubrication additives lubrication of automobile engine friction sliding interface, the wear rate and the coefficient of friction are greatly reduced. WS_2_ nanoparticles as grease additives in automotive transmission lubrication can prolong the service life of mechanical components and have a sealing effect and prevent material flaking [187]. Nickel-coated multi-walled carbon nanotubes (Ni-MWCNT) as engine oil additives can reduce the coefficient of friction and wear rate by 15–23% and 68–87%, respectively [188]. Nano-particles possess excellent physical and chemical properties, and when used as additives in gel-like lubricants, they can significantly enhance the lubricant’s extreme pressure and anti-wear performance. The mechanism by which nanoparticles form a protective film is known as the nano-repair mechanism. Materials such as nano-copper and silica can fill defects on friction surfaces through physical filling or chemical adsorption, thereby forming a continuous protective film and enhancing anti-wear and extreme pressure performance. Additionally, the “ball-bearing effect” is an independent rolling mechanism, and the synergistic action of the two effectively reduces the coefficient of friction. Research indicates that the use of nano-additives can reduce fuel consumption by 5–15%, reduce carbon deposits by 30–40%, and lower noise levels by 5–10 decibels, aligning well with the energy-saving and emissions-reduction requirements of the transportation sector. Furthermore, materials such as nano-copper and nano-silica achieve friction coefficient reduction and wear minimization by forming protective films on friction surfaces or through the “ball-bearing effect” mechanism [189].

Oil-soluble nano-copper-containing additives can repair worn surfaces, form composite films, and extend engine life [190]. High-speed rail gear box adopts grease containing nanographene, significantly reducing noise and temperature rise. Electric vehicle motor bearings use nano-graphene grease, which can both lubricate and export current to prevent galvanic corrosion damage. Railroad locomotive gearboxes use grease containing nano-copper additives with excellent friction reduction and anti-wear performance, and the nano-copper particles will form a layer of thin film with friction reduction and lubrication effect on the surface of friction parts, and at the same time, fill the microscopic craters to repair the micro-scratches on the surface of friction sub-surface, which significantly reduces friction and wear on friction sub-surfaces [191]. Yin [192] synthesized nano-grease by using domestic high-speed railroad axle box bearing grease as base grease and tungsten disulfide and silicon nitride nanoparticles as additives. The effects of tungsten disulfide and silicon nitride nanoparticles on the extreme pressure performance and anti-wear and friction reduction performance of the nano-grease were investigated. The results show that the lubrication mechanism of tungsten disulfide nanoparticles is self-repairing and film lubrication, and the lubrication mechanism of silicon nitride nanoparticles is self-repairing and ball-bearing-like, which is of certain reference value for the development of greases with excellent lubrication performance. The use of lithium composite grease containing nano-titanium dioxide, nano-silicon dioxide, nanoaluminum oxide and multi-walled carbon nanotubes as additives in racing engines can effectively improve the friction and wear performance of engines. Automobile transmissions use grease containing nano-copper, nanoaluminum, nanoalumina and nano-barium sulfate as additives, which can effectively reduce the coefficient of friction and wear rate of gears. Ali et al. [125] dispersed copper (Cu) and graphene (Gr) nanomaterials in 5W-30 fully synthetic motor oil at different concentrations, to investigate the mechanism of improving friction sliding in engines. Tribological results showed that compared to 5W-30, the lubrication of nano-additives reduced the wear rate (WR) and coefficient of friction (COF) by 25–30% and 26.5–32.6%, respectively. This is a promising method for improving the durability, service life, and fuel economy of friction sliding components in automotive engines. Automotive wheel hub bearings using grease containing additives such as nano-copper, nano-aluminum, nano-aluminum oxide, and nano-barium sulfate can effectively enhance bearing wear resistance and service life. Railway vehicle bogies using grease containing additives such as nano-copper, nano-aluminum, nano-aluminum oxide, and nano-barium sulfate can effectively reduce the friction coefficient and wear rate of the bogies. In research on optimizing the performance and enhancing the durability of automotive powertrain systems, core-shell micro- and nano-particles have demonstrated significant application value as novel lubricant additives. Automotive engines, during actual operation, must endure frequent cold starts, sudden acceleration/deceleration conditions that cause instantaneous high-load impacts, and prolonged low-speed, high-torque operation in urban traffic congestion. Additionally, they face challenges such as high temperatures in the combustion chamber and the oxidation and degradation of lubricating oil. Traditional lubricants struggle to meet the demands of modern engines’ long-term, high-intensity operation under boundary lubrication conditions, particularly in terms of film strength and stability. The unique core-shell structure of core-shell micro/nano particles endows them with excellent lubrication control performance: The rigid core, with its high hardness and strength, forms a load-bearing framework in critical friction pairs such as piston rings and cylinder walls, effectively dispersing contact stress and preventing lubrication film rupture. The flexible shell, based on strong interactions between surface-active groups and the metal substrate, rapidly constructs a boundary lubrication film with low shear strength at the friction interface through physical adsorption and chemical film formation mechanisms.

This section focuses on the application of nano-additives in transportation. Research shows that nano-additives effectively solve lubrication problems in transportation equipment under extreme conditions through multi-mechanism synergistic optimization, thereby extending equipment life and improving energy efficiency.

In summary, gel-like lubricant nano-additives are gaining increasing attention in the transportation industry. With their unique physical and chemical properties, various nano-additives can effectively function in different transportation scenarios. Incorporating these nano-additives into gel-like lubricants can significantly reduce the coefficient of friction, minimize wear, thereby extending the service life of mechanical components, improving operational efficiency, and simultaneously reducing energy consumption and environmental pollution. As the transportation industry increasingly demands efficient and environmentally friendly lubrication solutions, the application scope of gel-like lubricant nano-additives will continue to expand, effectively driving the industry toward sustainable development. However, the high cost of nano-materials remains a consideration, as even though the usage volume is small, the overall economic viability still requires careful balancing. If future large-scale production can reduce costs, the adoption rate of this technology will be significantly higher.

### 5.3. Special Area

In the aerospace, marine engineering and military industries, equipment is often required to operate in extreme environments, such as high vacuum, strong radiation, and corrosive environments [37]. These scenarios place demands on gel-like lubricants that exceed the capabilities of traditional materials. It is then necessary to improve the performance of conventional greases by adding suitable additives to match the scenarios in which they are utilized [193]. Under the stringent operating conditions of the aviation industry, core-shell micro- and nano-particles have emerged as highly promising lubricant additives due to their unique properties. With their distinctive core-shell structure, these particles feature a hard inner core that can withstand mechanical shocks caused by high loads, thereby maintaining the integrity of the lubricating film. Meanwhile, their flexible outer shell can rapidly form an adsorbed film on friction surfaces, effectively reducing the coefficient of friction and minimizing component wear. Li [194] studied the sealed bearings of aviation motors and utilized physical blending technology to compound two nanoparticles, Mullite and KH-mullite, as additives with polyurea grease respectively. The results show that when the concentration of Mullite is 0.03 wt%, the coefficient of friction and the diameter of the wear spot are the lowest, and compared with the basic grease, they are reduced by 9.3% and 11.1% respectively.The friction reduction and anti-wear performance of KH-mullite composite polyurea grease is better, and the coefficient of friction and the diameter of the wear spot are reduced by 22.4% and 15.6%, compared with that of the Mullite composite polyurea grease, and the friction coefficient and the diameter of the wear spot are reduced by 22.4% and 15.6%. KH-mullite composite polyurea grease has better friction reduction and anti-wear performance. Kanthavel et al. [195] studied aluminum ceramic composites with improved mechanical and chemical properties for aerospace and automotive applications. The combination of alumina and molybdenum disulfide to develop new aluminum composites was found to have significant improvement in tribological properties. A sliding distance of 1000 m, a sliding speed of 1.5 m/s, a minimum wear loss of 0.0102 g and a coefficient of friction of 0.117 were obtained when a load of 5N was applied. Molybdenum disulfide (MoS_2_) nanoparticles exhibit excellent tribological properties in high vacuum and intense radiation environments and can be used as aerospace lubricants [126]. Copper nanoparticles and SbS_4_ nanoparticles can fill pits on friction surfaces, repair cracks, and prolong the service life of equipment [196]. In the aerospace field, gel-like lubricant with nano additives can be used to lubricate helicopter main gearboxes, improving aircraft performance and reliability [197]. The high temperature and corrosion resistance of these materials make them widely used in marine environments and chemical equipment as well [127]. Du [198] prepared different grease samples with different nanoparticle additives, and the results showed that the composite lithium grease had the best tribological performance when the addition amount of compound nanoparticles was 1% and TiO_2_:CeO_2_ was 6:4, and its coefficient of friction and the diameter of the wear spot were reduced by 28.2% and 23.4%, respectively, compared to that of the original grease, and the roughness of the surface of the wear spot was reduced by 52.3%, and the maximum depth of wear mark was reduced by 58.9% compared to that of the base grease. The coefficient of friction and spot diameter are reduced by 28.2% and 23.4%, respectively, and the roughness of the spot surface is reduced by 52.3% and the maximum depth of abrasion by 58.9% compared with the base grease. The two nanoparticles were added together to provide a synergistic effect. However, there are some problems with the practical application of nanoparticles. For example, the long-term stability of nanomaterials needs to be evaluated by standardized tests, especially in shear stability and colloidal stability. Although the application in special fields has a high technical threshold, once the breakthrough is made, it may bring disruptive effects. The mass production of nanoparticles is currently facing two major challenges: process scaling bottlenecks and the absence of green synthesis standards. These issues severely hinder the sustainable development of the nanomaterials industry. In terms of process scaling, there is a significant gap between laboratory environments and industrial continuous production processes that is difficult to bridge. Under laboratory conditions, researchers can achieve near-perfect precise control over key parameters such as nanoparticle monodispersity and surface modification using sophisticated instrumentation and stringent operational conditions. Taking nanoparticle monodispersity as an example, by precisely controlling factors such as reaction temperature, concentration, and stirring speed, highly uniform nanoparticle dispersions can be prepared, ensuring that each particle has similar size and shape. In terms of surface modification, researchers can employ precise techniques such as layer-by-layer self-assembly and chemical bonding to impart specific functional groups to nanoparticles, thereby meeting diverse application requirements. However, when these successful preparation techniques are transferred to industrial production, they encounter numerous obstacles. Low production efficiency makes it difficult to ensure product consistency and stability.

The absence of green synthesis standards is equally concerning in the field of nanoparticle production, and as the application scope of nanomaterials continues to expand, this issue becomes increasingly severe. The preparation of metal nanoparticles requires extreme conditions such as high temperatures and pressures, as well as the use of large amounts of organic solvents and toxic reducing agents. These not only increase production costs but also cause severe environmental pollution. Furthermore, metal nanoparticles may be released into the environment during use, posing potential threats to ecosystems and human health. In order to effectively address these environmental risks, it is imperative to introduce life cycle assessment (LCA) standards to systematically evaluate the production process of nanoparticles. Process optimization can also be used to reduce costs, such as replacing solvent synthesis with mechanical chemistry. Continuous synthesis using reactors can be used for large-scale technological breakthroughs. Furthermore, comprehensive and objective evaluations of energy consumption, resource utilization, and environmental impact during the production process of nanoparticles should be conducted to provide scientific basis for optimizing production processes and reducing environmental risks.

This paper focuses on the application of nano-additives in extreme environments such as aerospace, marine engineering, and military industries. It demonstrates that nano-additives overcome the limitations of traditional lubricants through mechanisms such as core-shell structure synergy and layered lubrication, thereby enhancing tribological performance. However, challenges remain, including the absence of long-term stability testing standards for nano-materials, bottlenecks in mass production processes, and incomplete green synthesis standards. Overcoming these challenges will lead to disruptive changes.

In summary, the core value of gel-like lubricant nano-additives lies in enhancing stability under extreme conditions, significantly extending equipment lifespan, and reducing maintenance frequency [199]. In industrial manufacturing, it addresses lubrication challenges under high-temperature and high-pressure conditions; in transportation, it enables equipment to operate more quietly and efficiently under high-speed and high-temperature conditions; and in specialized fields, it performs exceptionally well under extreme environmental conditions. However, its widespread adoption is still hindered by technical challenges and economic considerations. Technologically, dispersion and stability require further optimization; economically, high costs limit its large-scale application. In the future, if breakthroughs can be achieved in preparation processes and cost control, the potential of this technology will be immeasurable.

## 6. Conclusions and Future Perspectives

### 6.1. Conclusions

With the continuous development of society, nanomaterials have become a key medium for breaking through the boundary of traditional material properties by virtue of their unique cross-scale interfacial effects and quantization properties [200]. As a revolutionary product in the field of lubrication materials, Nano additives in gel-like lubricants show subversive technological advantages in mechanical dynamics systems through the construction of their three-dimensional nano-topology, which can significantly reduce the coefficient of friction compared to traditional additives (corresponding to Section 4.2 of this paper). The extreme load carrying capacity is increased by 2–3 orders of magnitude (corresponding to Section 4.3). At the same time, the service life of mechanical components is extended by more than 30%. This technological breakthrough not only directly reduces equipment operation and maintenance costs, but also strategically aligns with global carbon neutrality goals, reducing energy loss by 8–12% per year for a single piece of industrial equipment.

This paper focuses on the different preparation methods of nano-additives in gel-like lubricants, primarily including physical preparation methods and chemical preparation methods. It then highlights the various categories of nano-additives in gel-like lubricants, summarizing metal-based nano-additives, metal-based oxide nano-additives, nano-carbon material additives, and other nano-additives. Next, the paper analyzes the anti-wear, friction reduction, oxidation resistance, and load carrying capacity of nano-additives in gel-like lubricants. It also explains the surface analysis techniques used. Subsequently, the paper introduces the various application scenarios of nano-additives in gel-like lubricants in industrial manufacturing, transportation, and special fields. Finally, it summarizes the existing issues and outlines future research directions. In summary, future experimental and theoretical research is still needed in the following areas:

(1) Unknown synergistic effect of composite nanoparticles

In recent years, researchers have studied different ratios of composite nano-additives to understand that they can significantly reduce the friction and wear of equipment. However, the mechanism of composite nanoparticles is insufficiently analyzed in depth, and the dynamic process of multi-component synergistic effect is still unclear. Therefore, in-depth research on the synergistic effect of composite nanoparticles is still needed.

(2) Insufficient dispersion stability

In high-temperature environments, nanoparticles in gel-like lubricating grease tend to agglomerate, resulting in insufficient dispersion and affecting lubrication performance. Although methods such as dispersants and online ultrasonic dispersion can improve dispersion, the dispersion effect is still unsatisfactory for different types of nano-additives and base oils. This requires solutions through surface modification and other technologies.

(3) Systematically study the influence of particle size and concentration gradients on the tribological properties of gel-like nano-lubricant additives; establish quantitative or semi-quantitative models/methods to evaluate the correlation between dispersion stability and tribological properties; use in-situ observation techniques to deeply explore the specific behavior of nanoparticles at the friction interface, the formation process, composition, and structural evolution of the protective layer, as well as its essential connection with particle parameters, dispersion, and surface interactions.

### 6.2. Future Prospects and Recommendations

In future research on gel-like lubricant nanoadditives, we recommend paying close attention to the following aspects.

(1) Driven by industrial sustainability goals, the research and development of gel-like lubricating materials should be improved in two ways. First, advanced processes such as microfluidic technology and efficient grinding should be adopted to achieve low-cost, large-scale production of materials, significantly reducing production energy consumption. Second, artificial intelligence-assisted molecular simulation technology should be used to optimize the microstructure of materials, and machine learning algorithms should be used to predict the optimal formula composition in order to overcome key technical bottlenecks such as the dispersion stability of nano-additives.
(2) The development of new gel-like lubricant nano-additives capable of withstanding extreme friction is of critical importance. Existing high-performance additives have significant limitations: their thermal stability is insufficient, with decomposition occurring at approximately 150 °C, leading to a sharp decline in lubrication performance; their flash points are generally below 100 °C, making them prone to volatilization or oxidation at high temperatures, which compromises safety and effectiveness; and their practical application range is narrow, being effective only at temperatures below 1000 °C and loads below 3000 N, failing to meet the stringent requirements of extreme environments such as aerospace, metallurgy and forging, and deep-sea drilling. Therefore, the future core research direction is to develop new additives with higher thermal stability, a broader operating temperature range, and stronger load-bearing capacity, particularly optimized for ultra-high temperature and ultra-high load conditions. This urgently requires a deep understanding of the synergistic mechanisms between additives and gel matrices, combined with material design, surface modification, and advanced preparation technologies, to drive performance breakthroughs and industrial applications under extreme conditions.
(3) Under the backdrop of stricter environmental regulations and sustainable development requirements, the development of gel-like lubricant grease nano-additives must balance performance with environmental sustainability. Traditional additives, which are difficult to degrade and prone to releasing harmful substances, pose threats to the ecological environment and human health. Therefore, the development of biodegradable formulations is urgent, requiring a focus on raw material selection, process optimization, and the use of green materials and low-energy synthesis methods. Additionally, as industrial equipment upgrades in areas such as smart manufacturing and new energy technologies advance, extreme operating conditions are placing higher demands on grease performance. The development of nano-additives must focus on innovative material design, microstructural optimization, and advanced preparation technologies to enhance wear resistance, oxidation resistance, and other properties, thereby ensuring the stable operation of industrial equipment and driving the green and intelligent transformation of manufacturing.

## Figures and Tables

**Table 1:** Comparison of traditional and nano additives in gel-like lubricants.

	Conventional Grease Additives	Nano Grease Additives
Particle size	Micron or larger particle size, relatively poor dispersibility, easy to agglomerate, difficult to enter the micro gap [6]	Nano-scale (1–100 nm), high specific surface area, good dispersion, can be uniformly distributed, easy to enter the tiny gap [15]
Tribological	Limited friction reduction, poor integrity and uniformity of the adsorption film formed [1]	Forms a more uniform, dense protective film that fills microscopic pits and scratches, significantly reducing the coefficient of friction [4]
Anti-wear	Weak protection against abrasion, reduced anti-wear performance in long-term use [1]	Good anti-abrasive properties, forming a tough protective film that reduces abrasive particles and adhesive wear [6]
Stability	Decomposes, oxidizes or deteriorates easily under extreme conditions (high temperature, high pressure, high shear) [1]	Better thermal and chemical stability to improve grease stability and reliability [2]

**Table 2:** Differences between physical and chemical methods for preparing nano-additives in gel-like lubricants.

Characterization	Physical Method	Chemical Method
Costs	Low (some methods) but high energy consumption (e.g., laser ablation) [37]	High (equipment, reagents, reprocessing) [38]
Technological complexity	Low (easy to operate, but difficult to control accurately) [39]	High (requires tight control of reaction conditions) [40]
Agricultural productivity	High (suitable for mass production) [41]	Low (partial method) [42]
Material properties	Low (wide particle size distribution, easily agglomerated, may introduce impurities) [42]	High (controlled size, shape, purity) [43]
Scope of application	Suitable for mass production of materials that do not require a high degree of precision [43]	Suitable for high-performance, high-precision materials (e.g., thin films, composites) [44]

**Table 3:** Nano-additives classification.

Type of Additive	Vantage	Areas of Application
Metal-based nano-additives	Self-healing strong high thermal conductivity [103]	Industrial machinery automotive field [104]
Metal Oxide Nano Additives	High chemical stability and high load capacity [104]	Aerospace marine equipment [42]
Carbon nanomaterial additives	Laminar lubrication mechanism High strength and thermal conductivity [104]	Precision Instruments New Energy Field [42]
Other nano additives	Filling special needs [105]	Extreme Environment Biomedicine Environmental Protection [105]

**Table 4:** Friction coefficient of nano-additives based on ISO standards.

Nano-Additive Category	Base Oil Type	Additive Concentration	Reduced Friction Coefficient
Graphene nanosheets [21]	Calcium-based gel-like lubricant	0.5–4 wt%	61%
Monodisperse silver (Ag) nanospheres [25]	Layered graphene sheets	0.1 wt%	40%
Nano-sized cerium oxide [61]	Lithium-based gel-like lubricant	0.6 wt%	28%
Zinc oxide-silicon dioxide core-shell composite nanoparticles [62]	Lithium-based gel-like lubricant	1 wt%	18%
TiO_2_ and CeO_2_ composite nanoparticles [63]	Composite lithium-based grease	6:4	30.5%
Multi-walled carbon nanotubes (MWCNT) and aluminum oxide (Al_2_O_3_) [78]	Lithium-calcium-based gel-like lubricant	4 wt%	Lowest coefficient of friction
MoS_2_/AlOOH nanocomposite [122]	PAO_4_ Base Oil	0.5 wt%	50.47%
Tubular graphite carbon nitride [123]	Lithium-based grease	0.06 wt%	10%
Graphene nanosheets [124]	Composite lithium-based grease	0.15 wt%	32.8%
Copper and graphene nanomaterials [125]	motor oil	0.4 wt%	26.5–32.6%
Mullite [126]	Polyurea grease	0.03 wt%	15.6%
1% TiO_2_:CeO_2_ [127]	Lithium-based grease	6:4	28.2%

**Table 5:** Wear scar diameter of nano-additives based on ISO standards.

Nano-Additive Category	Base Oil Type	Additive Concentration	Diameter of Wear Scar
Graphene nanosheets [21]	Calcium-based gel grease	4 wt%	45%
Nano Al_2_O_3_, nano ZnO [59]	Gel-like lubricant	Al_2_O_3_ content is 0.4 wt%, ZnO content is 0.6 wt%	28%
Nano-sized cerium oxide [61]	Lithium-based gel-like lubricant	0.6wt%	13%
TiO_2_ and CeO_2_ composite nanoparticles [63]	Composite lithium-based grease	6:4	29.2%
Multi-walled carbon nanotubes (MWCNT) and aluminum oxide (Al_2_O_3_) [78]	Lithium-calcium-based gel grease	4 wt% ratio	Minimum scratch diameter
Graphene oxide (GO) and reduced graphene oxide (RGO) [83]	Graphene flakes	4 wt%	70%
MoS_2_/AlOOH nanocomposite [122]	PAO_4_	0.5 wt%	42.34%
Tubular graphite carbon nitride [123]	Lithium-based grease	0.06 wt%	28%
Ag/MWCNT nanocomposites [140]	Motor oil	0.18 wt%	32.4%

**Table 6:** Load capacity of nanoadditives based on ISO standards.

Nano-Additive Category	Base Oil Type	Additive Concentration	Load Capacity
Zinc oxide nanoparticles [27]	Lithium-based, composite lithium-based, and polyurea greases	0.6 wt%	Enhanced load-bearing performance of grease
Graphene oxide and zinc oxide [35]	Palm greases	0.5 wt%	Improved by 30% and 60% respectively
Zinc oxide-silica core-shell composite nanoparticles [62]	Lithium-based gel greases	1 wt%	Enhanced load-bearing capacity of interface lubricating film

**Table 7:** Comparison between traditional grease and gel-like lubricant.

Situation	Conventional Grease Disadvantages	Advantages of Gel-like Lubricant
Metallurgical mill gearboxes [3]	High temperature oxidation, carbon buildup	High temperature self-repairing and wear reduction
Injection molding machine screw [4]	Oil carbonization, contamination	Resistant to high-temperature oxidation and prolonged life
Industrial Robot Reducer [1]	Shear failure and loss of precision	Shear resistant, high cleanliness
Wind Turbine Main Shaft Bearings [6]	Salt spray corrosion, maintenance difficulties	Corrosion-resistant and maintenance-free for long periods
High-speed CNC machine tool spindles [183]	Grease splattering, thermal deformation	Resistant to centrifugation and low temperature rise

## Data Availability

Not applicable.
